# Supplementary material for: Genomic analyses in Drosophila do not support the classic allopatric model of speciation
Source: Evol Lett. 2026 Jan 15;10(2):186–94. doi: 10.1093/evlett/qraf053 (PMC13043940; doi:10.1093/evlett/qraf053)
Supplement: qraf053_Supplemental_File [file qraf053_supplemental_file.pdf]

Supporting Information for

Genomic analyses in *Drosophila* do not support the classic allopatric model of speciation.

Leeban H. Yusuf[1], Dominik R. Laetsch[2], Konrad Lohse[‡,2], Michael G. Ritchie[‡,1]

Email: ly36@st-andrews.ac.uk

This PDF file includes:

Supporting text
Figures S1 to S15
Tables S1 to S6
SI References

Supplementary Text

1.1 Data sampling and quality control.

To infer the demographic histories of species pairs of *Drosophila*, we collated data on reproductive isolation (RI), range overlap and genetic distance for *Drosophila* species pairs originally collated by Coyne and Orr (*1*, *2*), and expanded by Yukilevich (*3*, *4*). This represents the largest collection of RI measurements in *Drosophila*. As in (*1*, *2*), and other comparative surveys of *Drosophila* (*3*–*7*), we refer to "species pairs" as closely related but not necessarily independent pairs of species (i.e. our dataset consisted of 93 species pairs involving 58 species). Our goal was to infer speciation histories for as many species pairs found in (*1*, *2*) as possible to ensure results were comparable with previous comparative analyses. Briefly, Coyne and Orr (*1*, *2*) calculated premating isolation for each species pair as the percentage of successful copulations for homotypic and heterotypic matings during no-choice, single-choice, or multiple-choice mating experiments. Postzygotic isolation was calculated as the percentage of F1 hybrid males and females that were sterile or inviable. For each reciprocal mating, measures ranged from 0 (F1 hybrids are viable and fertile) to 1 (all hybrids produced are either sterile or inviable). The overall postzygotic isolation index is the average of both reciprocal mating interactions. For detailed descriptions of RI and range measures see references (*3*, *4*).

We sought to combine data on RI and range overlap with inferences of the demographic history of species divergence based on whole genome sequence (WGS) data. For each focal species pair, our data consisted of a single reference genome assembly as well as additional short read WGS data for a single sample of each species. Both were obtained from publicly available datasets via NCBI. 22 of the reference assemblies were based on long read data that were generated as part of large-scale efforts to produce highly contiguous *Drosophila* genome assemblies (*8*) (see Supplementary Table 1-3); the remaining 8 assemblies were based on Illumina data. For species pairs for which multiple genome assemblies were available, we assessed genome quality (using BUSCO v5.1.2 single-copy completeness) and selected the genome with the highest BUSCO score (>90% single-copy completeness) (*9*) as the reference for each pair.

To standardize quality of WGS datasets, we filtered the possible sequencing datasets for each species (see Supplementary Table 1 for a list of samples used): specifically, we restricted our analyses to high coverage datasets (i.e. total number of bases between 1 and 50 Gb) and removed pool-sequenced samples or samples that had been experimentally manipulated before sequencing. We selected data from wild-caught individuals where possible. For each focal species, we randomly sampled a single WGS dataset using a custom R script from the list of publicly-available samples. The randomly sampled WGS data for each pair were downloaded using the sratools functions pre-fetch v2.11.0 and fasta-dump v2.11.0.

1.2 Genome annotation, mapping and variant calling.

Reference genomes were annotated in the same way to minimize differences in gene prediction. Annotation was performed for all genome assemblies using BRAKER2 v2.1.6 (*10*). We used only *D. melanogaster* (Flybase v.6.39) proteins as evidence for all annotations. Subsequently, we extracted intronic regions from each genome annotation using BEDOPS v2.4.39 and bedtools v2.30.0 (*11*, *12*).

All reads were trimmed using fastp v.0.20.1. The reference genome assembly for each pair was indexed (Supplementary Table 3) (*13*, *14*). Species-specific paired-end or single-end read WGS data were then mapped to pair-specific genome assemblies using bwa-mem2 (v. 2.2.1) for species with paired end reads and bwa-mem (v. 0.7.17) for species with single end reads (*13*, *14*). For 9 species pairs a representative genome for mapping and variant calling did not exist for either species. For these pairs we used a reference genome from a closely related outgroup species. Reads were sorted, duplicates mapped and read groups added using sambamba v. 0.8.1 and picard v2.26.3(*15*, *16*). Trimming and mapping were implemented in the workflow engine

Snakemake v. 6.5.1 (*17*). Variants were called for reads in each pair using Freebayes v1.3.5 and subsequently filtered for missing genotypes, read mapping bias (RPL<1 | RPR<1 | SAF<1 | SAR<1), and a minimum read depth of 2, using bcftools v1.11 and gIMble v.0.6.1 (*18–20*). We refer to pair-specific datasets which have been filtered as above as 'callable and filtered' datasets. The workflow for the filtering process is summarized in Supplementary Figure 9.

1.3 Sampling intronic blocks.

To generate comparable datasets for demographic modelling across all pairs, we sampled intronic blocks from pair-specific, callable and filtered datasets. Our rationale for using intronic blocks to model speciation history (Supplementary Figure 9), was to maximise information contained in a single genome per species: our inference scheme summarizes variation in short sequence blocks that are assumed to be non-recombining and to evolve neutrally. Thus, coding regions are not suitable for such block-wise inference given their functional constraint. Similarly, although intergenic regions show, on average, similar levels of sequence conservation to introns in *Drosophila*, distinguishing conserved from selectively-neutral intergenic regions is challenging and relies heavily on accurate annotation (*21–23*). In contrast, annotation of genic regions and introns is comparatively easier and more reliable across pairs. Since functional constraint varies across introns in *Drosophila*, we applied additional filters to the filtered callable datasets (*21, 23*). Specifically, first introns were removed because they evolve more slowly than non-first introns (*21*), likely owing to their higher abundance of regulatory elements. We also removed the first and last 10 bases from all introns in our analyses using bedtools v2.30.0, and a custom python script.

We intersected filtered intronic sequence with callable positions using bedtools intersect v2.30.0. To minimise the effect of linkage between blocks on our analysis, each gene could only contribute a single intronic block to each pair-specific dataset. In practice, however, for intronic sequence to be useable it also had to satisfy the filtering criteria detailed in subsection 1.2 and be sufficiently long; this meant that not all genes contributed an intronic block to the dataset for each pair. Intronic blocks for every species pair were also manually checked to confirm that only a single intronic block per gene was sampled. To confirm that our datasets of filtered intronic blocks were more selectively neutral than the background, we compared absolute genetic divergence d_{XY} calculated across filtered introns to d_{XY} across all callable sites in the genome. As expected, given the near absence of functional constraint in filtered introns, d_{XY} in filtered introns was higher than genome wide d_{XY} in all pairs (Supplementary Figure 10).

For each species pair, short blocks were partitioned using gIMble v.0.6.1. For every pair, the block length was chosen to give an average of three pairwise differences (pair-specific block length = $3/d_{XY}$) between species per block. Species pairs with fewer than 250 blocks and pairs for which the short intronic blocks had higher within-species heterozygosity compared to fixed differences between species were removed from the analysis. These two filters removed 9 species pairs from the analysis. For the remaining 93 pairs, block sizes ranged from 60 – 341 bases, and the number of blocks ranged from 367 – 6390 (Supplementary Table 2). The final datasets consisting of pair-specific, filtered, short intronic blocks were converted into blockwise site-frequency spectra (bSFS), containing blockwise counts of all possible mutational configurations, using gIMble (v.0.6.1) which were further summarised into S distributions by assigning heterozygous sites to haplotypes at random assuming binomial sampling (see Methods). To account for differences in the number of blocks between species pairs, we normalised each S distributions by $500/n_i$ (where n_i is the number of blocks in pair i). This is analogous to taking an average across all subsamples of 500 blocks for each taxon pair.

1.4 Modelling gene flow and divergence.

To understand demographic histories and the degree of gene flow for species pairs, we fitted a range of models of demographic history to the S distribution of each species pair : (a) **a strict isolation (SI) model** (most consistent with *strict* allopatric speciation), i.e. an instantaneous split of an ancestral population of effective size N_e at time T_0 without gene flow, (b) **isolation with**

migration (IM), where an ancestral population diverges with symmetric migration ($M = 4N_em$ migrants per generation) between the time of divergence and the present, (c) an **isolation with initial migration model (IIM)**, where an ancestral population diverges with an initial period of symmetric migration and gene flow ceases at T_1 and (d) **a secondary contact (SC) model**, where an ancestral population diverges in allopatry, but experiences an instantaneous recent, pulse of gene flow, i.e. a total proportion (*f*) of the population is introgressed at time T_1. In all cases, we assumed a single N_e parameter that is shared between the ancestral population and the daughter species. Time parameters are scaled in units of $2N_e$ generations. See Supplementary Figure 7 for visualisations of each model.

1.4.1 Evaluating difference in support for gene flow between pairs based on current range overlap.

Statistical analyses were performed in R using the package stats v.4.0.2, and in *Mathematica* (*29*). We hypothesised that if current range overlap is predicted by the demographic history of speciation, sympatric pairs should (1) fit an IM model better than an SI model more often than allopatric pairs, and (2) show greater support for an IM model over an SI model compared to allopatric pairs. We tested these two predictions by calculating the difference in log-likelihood (ΔlnCL) between the IM model and SI model. Pairs showed support for an IM model over an SI model if ΔlnCL was larger than the critical threshold ($p < 0.05$). We compared the number of allopatric and sympatric pairs fitting an IM model over an SI model using Fisher's exact test. To evaluate whether there was a difference in average support (ΔlnCL) for an IM model over an SI model between allopatric and sympatric pairs, we used *Mann-Whitney U* tests. This framework was used to test the difference in model support between allopatric and sympatric pairs, and difference in the proportion of pairs fitting a given model over another.

We used *Mann-Whitney U* tests to assess differences in the estimates of the scaled migration rate (M) between allopatric and sympatric pairs across models with gene flow (IM, IIM, SC). Additionally, we computed the probability that an individual lineage is involved in gene flow as $1 – e^{-\tau 0 * M}$ under the IM and IIM model. This allows comparisons of M estimates obtained under the IM and IIM model with the admixture fraction (*f*) computed in our SC model, which is already a per lineage probability. Similarly, we asked whether currently allopatric and sympatric pairs differed in the average probability of migration and/or the scaled divergence times and effective population sizes using *Mann-Whitney U* tests.

1.4.2 Assigning a best-fitting model to each species pair.

To assign the best-fitting model to each species pair, we compared model support pairwise between competing models. Critical values for the difference in support (ΔlnL) between nested models (at a significance level of 0.05) were determined assuming a χ^2 distribution, which is conservative. For example, the SI model was considered the best-fitting model for pairs that did not fit an IM model significantly better. Conversely, pairs that fit an IM model better than an SI model, but did not show significant support for an IIM or SC model over the IM model, were assigned the IM model as best-fitting. Pairs that fit an IIM model significantly better than an IM model—and for which the IM model fit significantly better than the SI model—were assigned the IIM model as best-fitting. However, only one pair fit an IIM model better than an IM model.

To assign pairs to the secondary contact (SC) model—which is both more complex than the IM model and non-nested within it—we accepted it as the best-fitting model only when relative model support (ΔlnCL) exceeded the critical value for SI vs. SC (ΔlnCL > 5.991, 2 d.f.) **and** showed greater relative support than when compared against the IM model (ΔlnCL > 3.841, 1 d.f.). We confirmed that pairs best fitting the SC model displayed a higher frequency of blocks with few or no differences, consistent with a signal of recent gene flow (Supplementary Fig. 15).

1.5 Simulations – examining the effect of realistic recombination rates on model fit.

To understand how our inference based on the S distribution is impacted by recombination within blocks and to test whether the pervasive signal of long term gene flow could be an artefact of ignoring recombination within blocks, we used *msprime* (*27*, *28*) to simulate blocks of the same size as our empirical dataset (Supplementary Table 2) under the best fitting SI history for each species pair. For every taxon pair we simulated data without recombination and with recombination assuming a fixed rate of ρ = 1.03 cM/Mb, half the average recombination in *D. melanogaster* females to account for the achiasmy in males (*26*). Additionally, we assumed a per generation mutation rate of 3.32 x 10^{-9} as mentioned above. For every pair, we simulated 2,000 blocks and normalised the S distributions as described above.

To quantify the bias in MLEs of parameters and to determine the false positive rate of model selection with and without recombination, we fitted simulated S distributions for every pair to the SI and IM models and assessed the difference in log-likelihood as detailed above for the empirical data. We simulated two pseudo-observed datasets (PODS): (1) without recombination and (2) with a realistic recombination rate and asked i) whether there is significant support for an IM relative to an SI model and ii) how rates of gene flow inferred from simulated data compare to empirical estimates.

When simulating blockwise data under an SI history and no recombination, parameter estimates closely match the true values and none of the PODS (0 out 93 species pairs) erroneously fit an IM model over a SI model (Supplementary Figure 8). This is consistent with the expected false positive rate (FPR) given the χ^2 test we performed on ΔlnL. In contrast, when simulating under a SI model with recombination, 24 out of 93 PODS erroneously support an IM over an SI model which corresponds to a FPR of 25%. However, comparing the relative support (ΔlnL) between SI and IM for PODS (simulated under SI with recombination) with the empirical estimates shows that the IM model has considerably higher support in the real data compared to the PODS simulated without gene flow (two-sample Kolmogorov-Smirnov test: D = 0.79, p = <0.001).

Furthermore, we investigate the impact of recombination on our inference by comparing scaled rate of gene flow (M) and divergence time (T_0) estimates for PODS simulated under a SI model with recombination, with empirical parameter estimates. In the 77 species pairs that best fit an IM model in the empirical data the M estimates for the corresponding PODS (simulated with recombination under SI) are much lower than the empirical estimates (two-sample Kolmogorov-Smirnov test: D = 0.45, p= <0.001). Similarly, estimates for T_0 (two-sample Kolmogorov-Smirnov test: D = 0.58, p= <0.001) are also considerably lower for data simulated under the best fitting SI model compared to the empirical estimates across the 77 pairs. Thus, we conclude that while plausible rates of recombination may result in erroneous inference of gene flow, this effect is subtle and can neither explain the amount of gene flow we infer, nor the strength of support for speciation histories that involve gene flow that we find in the empirical data.

1.6 Understanding the effect of non-independent pairwise sampling on modelling gene flow.

To maximise the overlap with seminal comparative surveys of RI, our dataset includes non-independent species pairs. Assessing the impact of phylogenetically non-independent sampling on our main result is important because historic gene flow between a single pair of ancestral taxa may contribute to gene flow signals between in several focal species pairs. To understand the effect of the pseudoreplication, we used a pruned phylogeny of *Drosophila* inferred using single-copy orthologues from Kim et al. (2024) *(31)*, to trace each pairwise comparison of species back to its most recent common ancestor in the species tree. We quantified the number of pairwise comparisons that could be traced back to each speciation event (Supplementary Figure 11) and quantified average support for alternative models by averaging the difference in log-likelihood

between nested models across all pairs corresponding to each node. Additionally, we also quantified node-level estimates for gene flow, divergence time and N_e by averaging parameter estimates for all pairs assigned to an ancestral node. This simple node-level correction approach reduced our dataset to 36 observations across the *Drosophila* phylogeny.

Of the 36 node-level observations across our dataset, 31 nodes show evidence of significant support for an IM model over a strict isolation model (Supplementary Figure 12). Similarly, of the 36 nodes in the phylogenetically corrected dataset, 31 nodes have non-zero probabilities of migration (mean probability of migration = 0.68). Mean estimates of ancestral effective population (N_e = 858,000) and divergence time (T_0 = 5,964,000) in our phylogenetically corrected dataset strongly resemble mean scaled effective population size (N_e = 888,000) and divergence time (T = 4,917,000) estimates. This suggests that the overwhelming signal of gene flow identified across pairwise comparisons is not inflated because of non-independent sampling. Additionally, in the corrected dataset, we found only one node (*D. athabasca – D. affinis*) where the IIM model fits significantly better than an IM model. However, we note that at some ancestral nodes, node-based estimates are heavily influenced by one or two species (e.g., *D. melanogaster* in the *melanogaster* group). Importantly, this not only reflects a bias in our genomic dataset but in the original comparative surveys of reproductive isolation which motivated our choice of taxa.

1.7 Sensitivity analyses

For a number of pairs best fitting an IM model, parameter estimates for migration (M) and divergence time (T_0) hit the upper bounds, implying that for these pairs parameter estimates are unreliable. To assess the robustness of our main results, we removed pairs for which M was found to be larger than 10 (n=8) and T_0 larger than 9 (n=14), resulting in a reduced dataset of 71 pairs. We assessed again whether there was a difference in the probability of gene flow (two-sample Wilcoxon tests: W = 603, p = 0.847) and scaled divergence time (two-sample Wilcoxon tests: W = 683, p = 0.471) between sympatric and allopatric pairs with this reduced dataset only containing taxon pairs with reliable parameter estimates. As with our full dataset, we found no difference in the probability of migration and divergence times between allopatric and sympatric pairs.

Supplementary Figures

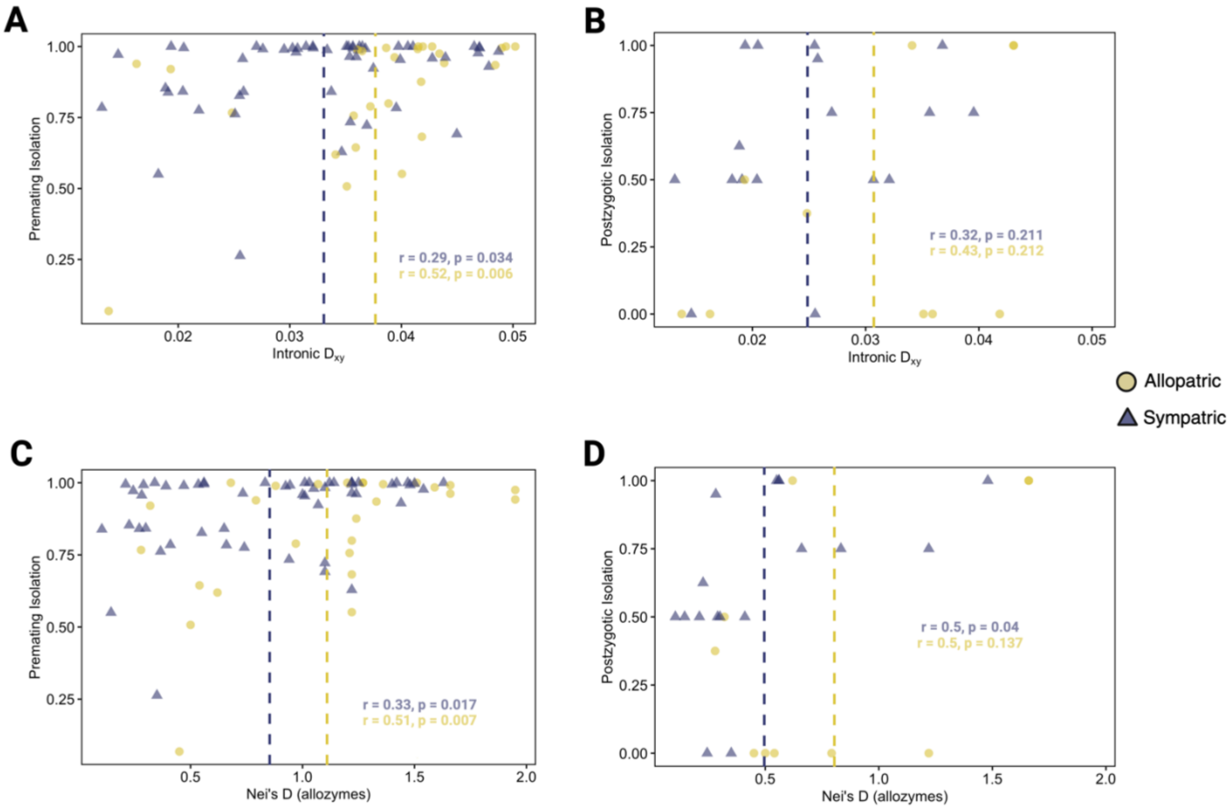

Supplementary Figure 1: Relationship between d_{xy} (absolute genetic divergence in filtered introns) and measures of reproductive isolation. Genetic divergence calculated based on short intronic blocks sampled from whole-genome data in this study (A and B), and previous estimates of genetic divergence (Nei's D) based on allozymes (C and D). Dashed lines on the top two panels indicate mean intronic d_{xy} (A and B) for allopatric and sympatric species pairs, and dashed lines in the bottom two panels indicate mean Nei's D calculated from allozymes for allopatric and sympatric pairs. A and C show estimates of premating isolation on the y-axis, and B and D show estimates of postzygotic isolation taken from previous comparative surveys of *Drosophila*. Pearson correlation coefficients and p-values for the relationship between measures of RI and measures of genetic divergence for sympatric and allopatric pairs are shown in each panel.

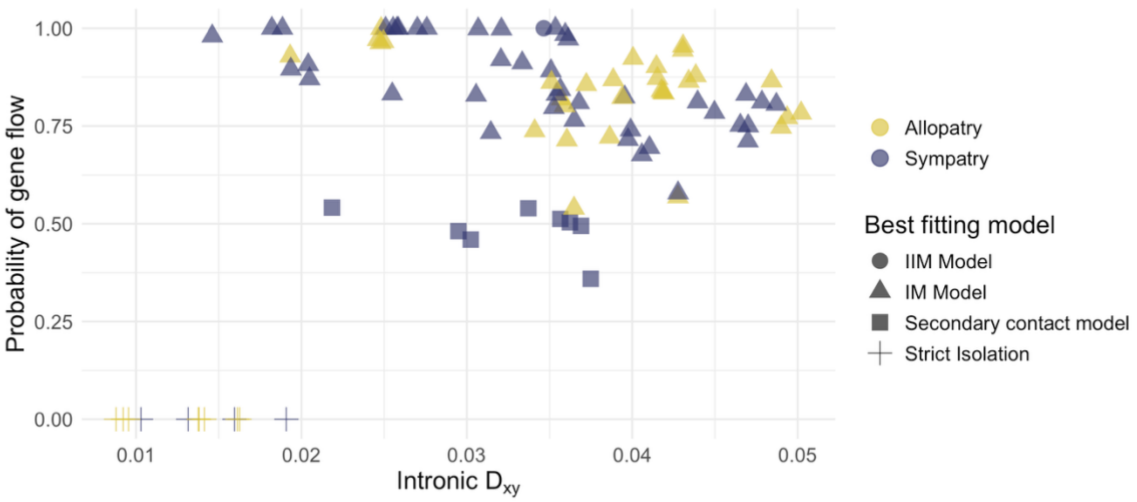

Supplementary Figure 2: Relationship between d_{xy} (absolute genetic divergence in filtered introns) and probability of gene flow based on the best-fitting model. Pairs either fit an IM model with continuous gene flow, a secondary contact model with a burst of gene flow or a strict isolation model best (denoted by different shapes). Biogeography (whether pairs are currently allopatric or sympatric) is denoted by colours. The MIG model contains an infinite period of continuous gene flow and no definable divergence parameter, thus it is not possible calculate a probability of long-term migration for the one pair that showed best support for the MIG model.

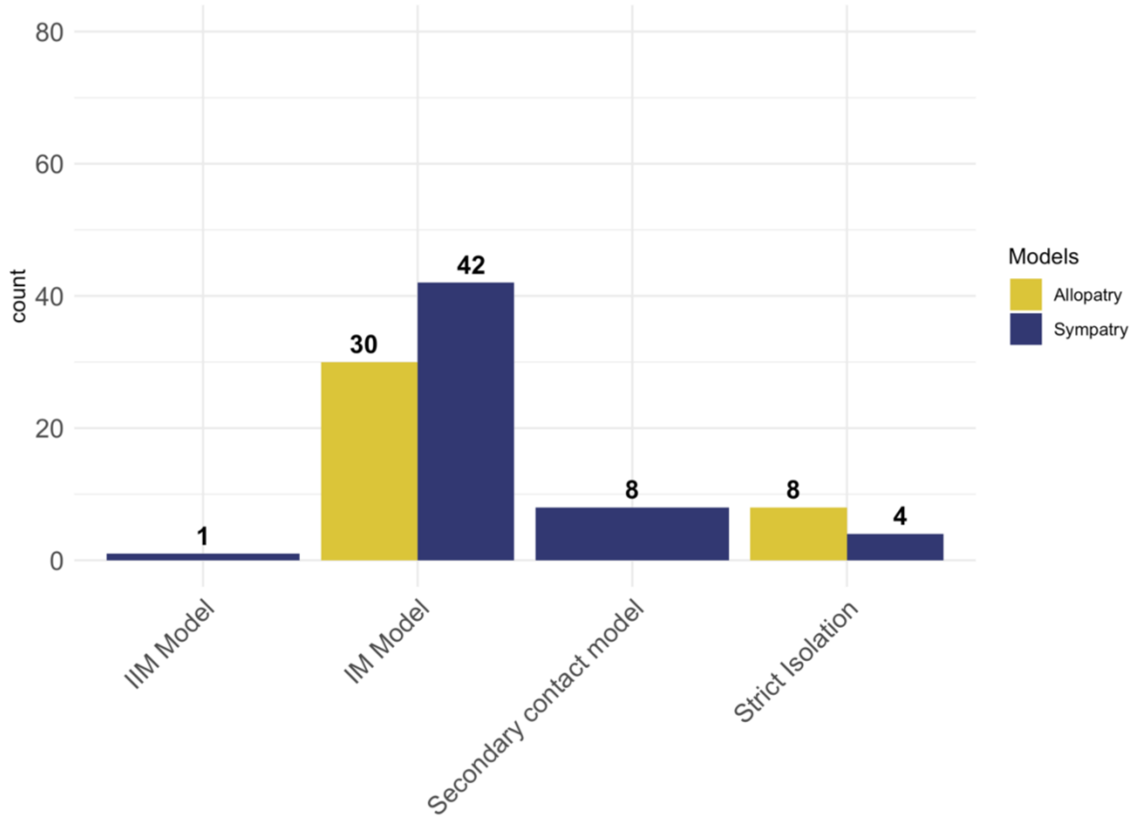

Supplementary Figure 3: Pairs and their best-fitting models. Pairs categorised into allopatry and sympatry, and the values on top of bars represent the number of allopatric and sympatric pairs best-fitting one of the three models. We found no allopatric pairs that best fit an SC or IIM model, so these are not represented here.

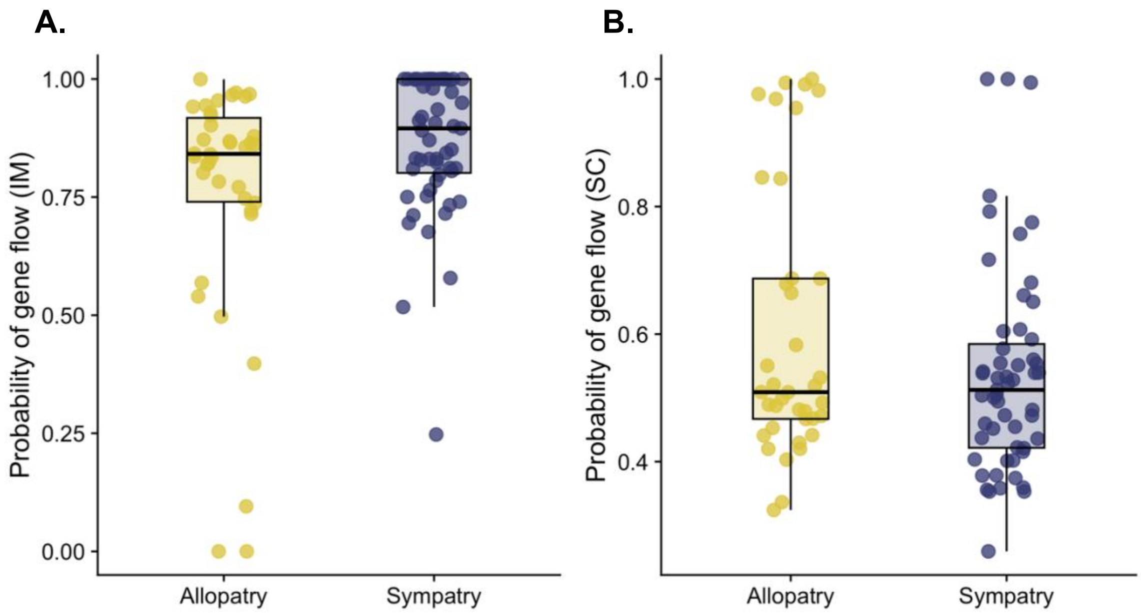

Supplementary Figure 4: Probability of gene flow under different demographic histories of species divergence for all pairs. For A and B, plots compare the probability of a single lineage migrating. A) Sympatric pairs are inferred to have a marginally higher mean probability of gene flow during a continuous migration phase than allopatric pairs under an IM model. B. Similar levels of inferred gene flow are seen during a short admixture phase under a secondary contact scenario.

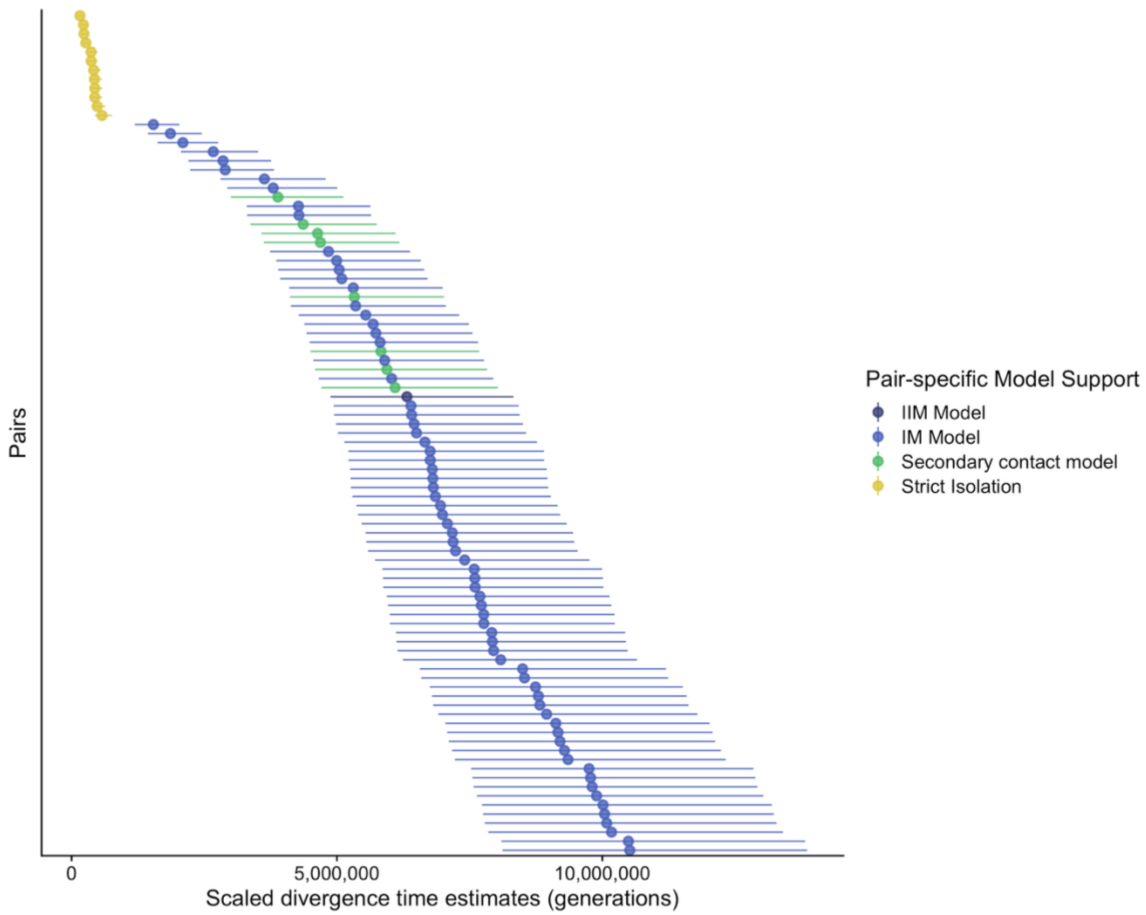

Supplementary Figure 5: Initial divergence time estimates for *Drosophila* pairs under the best-fitting models. Parameter estimates (T) were converted into generations using a *D. melanogaster* mutation rate of 3.32 x 10^{-9} (*26*), represented by the circles. Upper and lower bound of divergence time for each pair reflect the upper and lower bounds of the *D. melanogaster* mutation rate (upper: 2.52 x 10^{-9}; lower: 4.30 x 10^{-9}). Pairs are coloured by their best-fitting model.

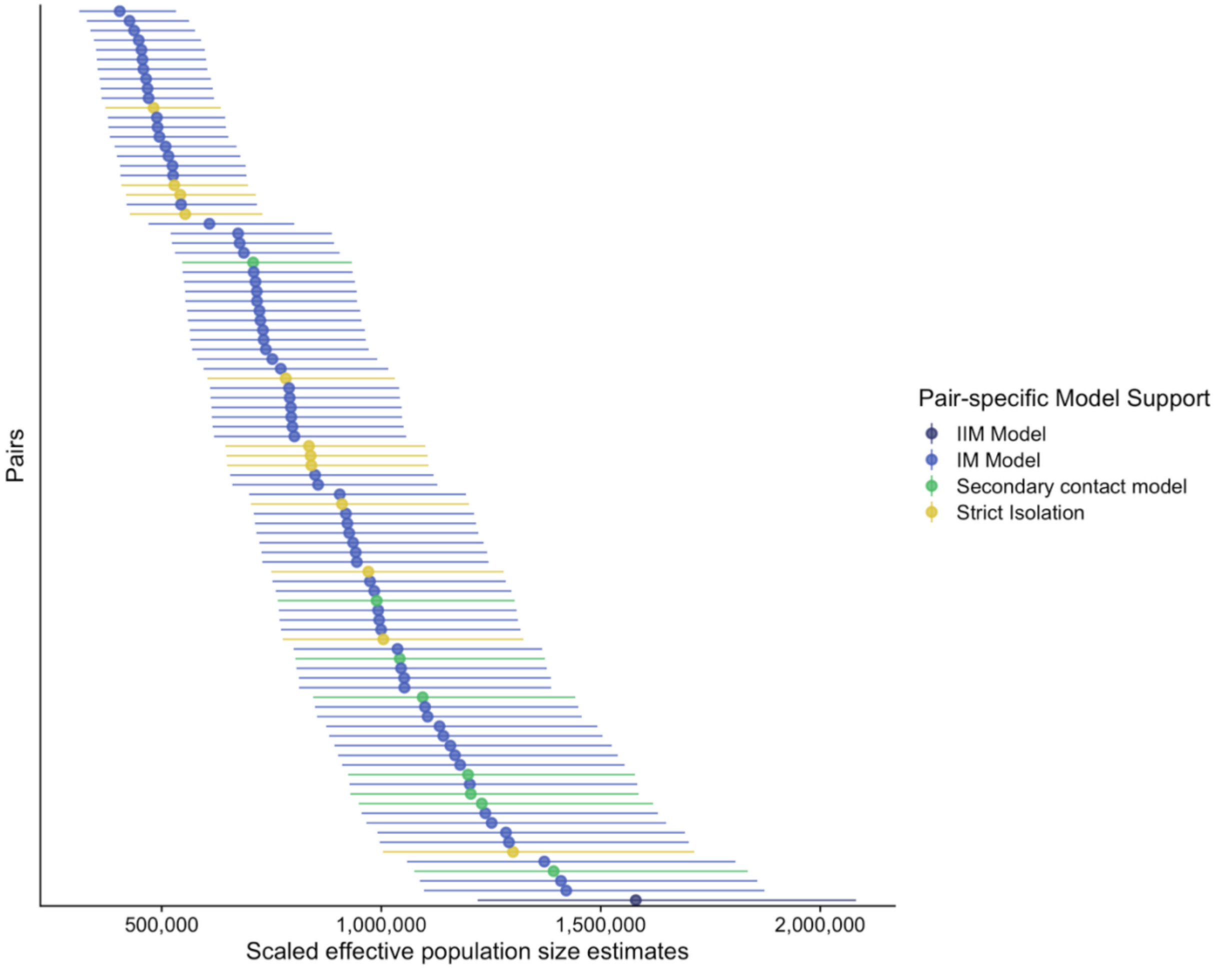

Supplementary Figure 6: Estimates of the effective population size (N_e) for Drosophila pairs under the best-fitting model. Parameter estimates were scaled using a *D. melanogaster* mutation rate of 3.32×10^{-9} (*26*). Upper and lower bounds reflect the upper and lower bounds of the *D. melanogaster* mutation rate (upper: 2.52×10^{-9}; lower: 4.30×10^{-9}). Pairs are coloured by their best-fitting model.

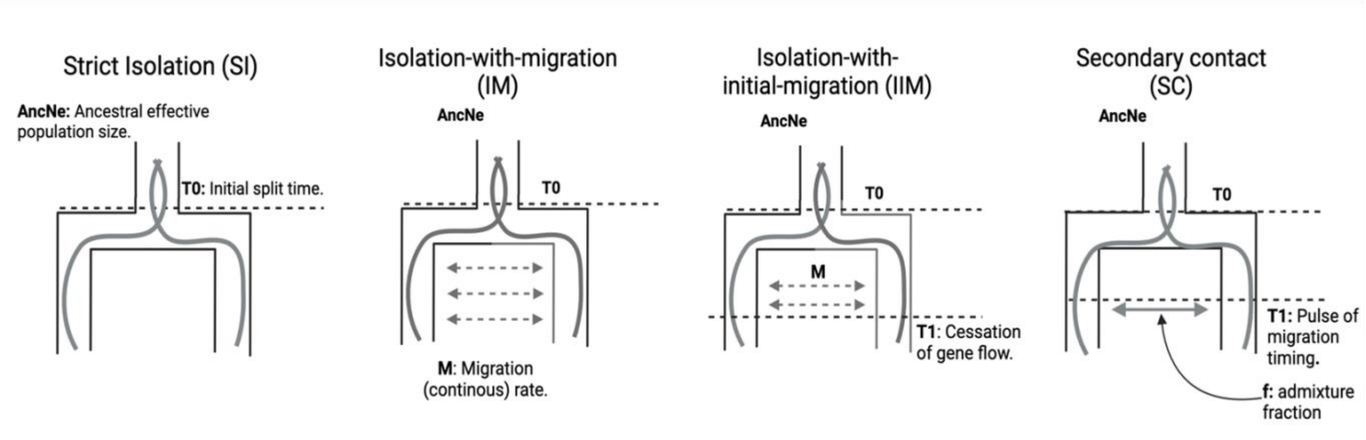

Supplementary Figure 7: Speciation models considered in the analysis. Models include a Strict Isolation (SI) model representing allopatric speciation without any gene flow (2 parameters), an isolation-with-migration (IM) model with continuous gene flow (3 parameters), an isolation-with-initial-migration model (IIM) which includes an initial phase of migration followed by no gene flow (4 parameters), and a secondary contact (SC) model which includes a recent pulse of migration (4 parameters).

277

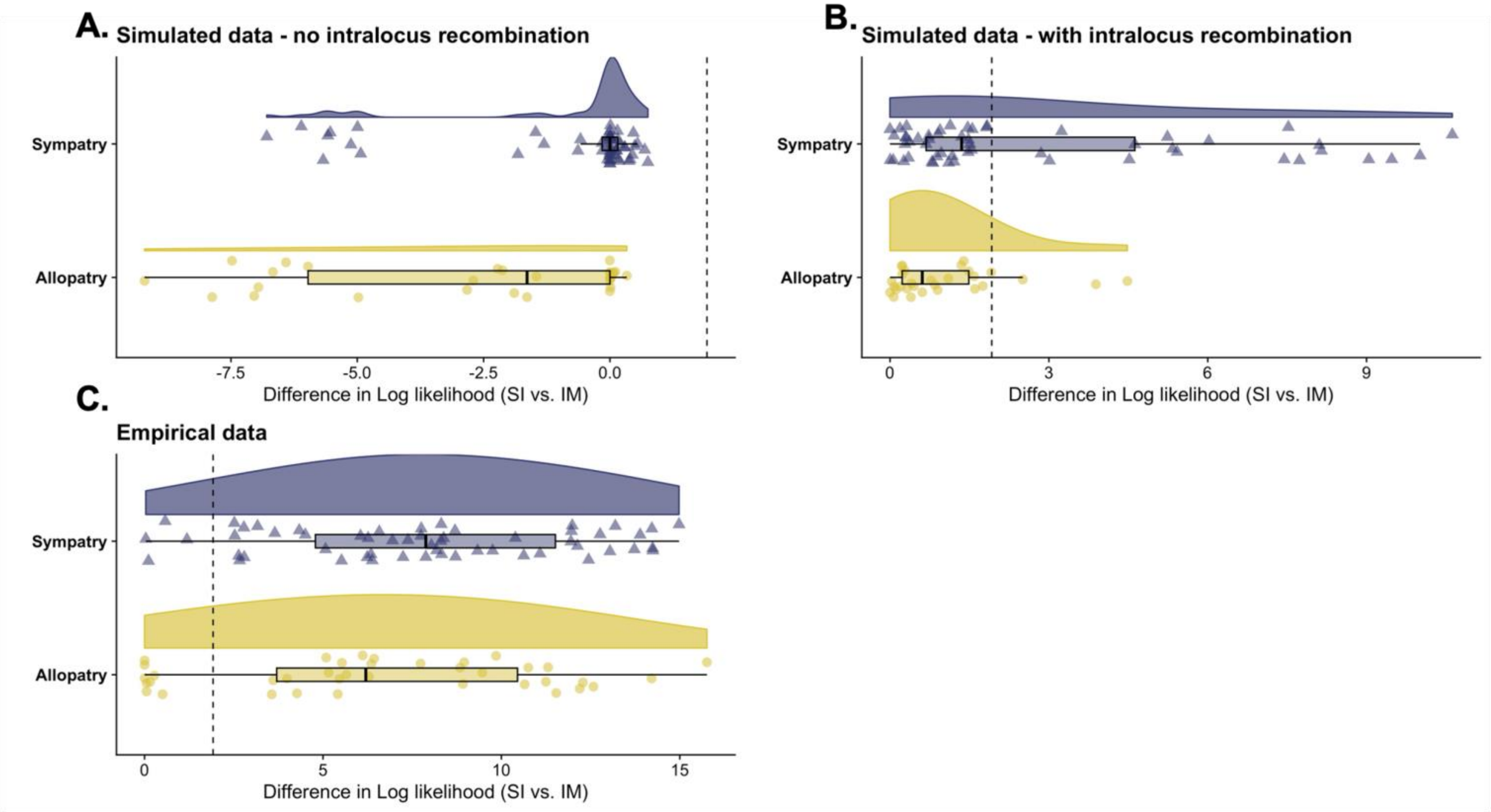

Supplementary Figure 8: Comparison of the difference in log-likelihood between a strict isolation (SI) model and an isolation-with-migration model (IM) between **A.** Data simulated according to optimized strict divergence history parameter estimates but with no recombination, **B.** Data simulated under an optimized strict divergence history with D. melanogaster levels of recombination, and **C.** empirical data. The dashed line indicates the critical threshold ($p < 0.05$). **A.** Simulations with no recombination show a false positive rate of detecting a history of speciation-with-gene-flow where there is none is 0%. **B.** Simulated data with realistic levels of recombination show a higher false positive rate of 25%. Comparison of **B.** and **C.** demonstrate that simulations with recombination do not recapitulate as strong support for a speciation history with gene flow across allopatric and sympatric pairs as the empirical data.

278

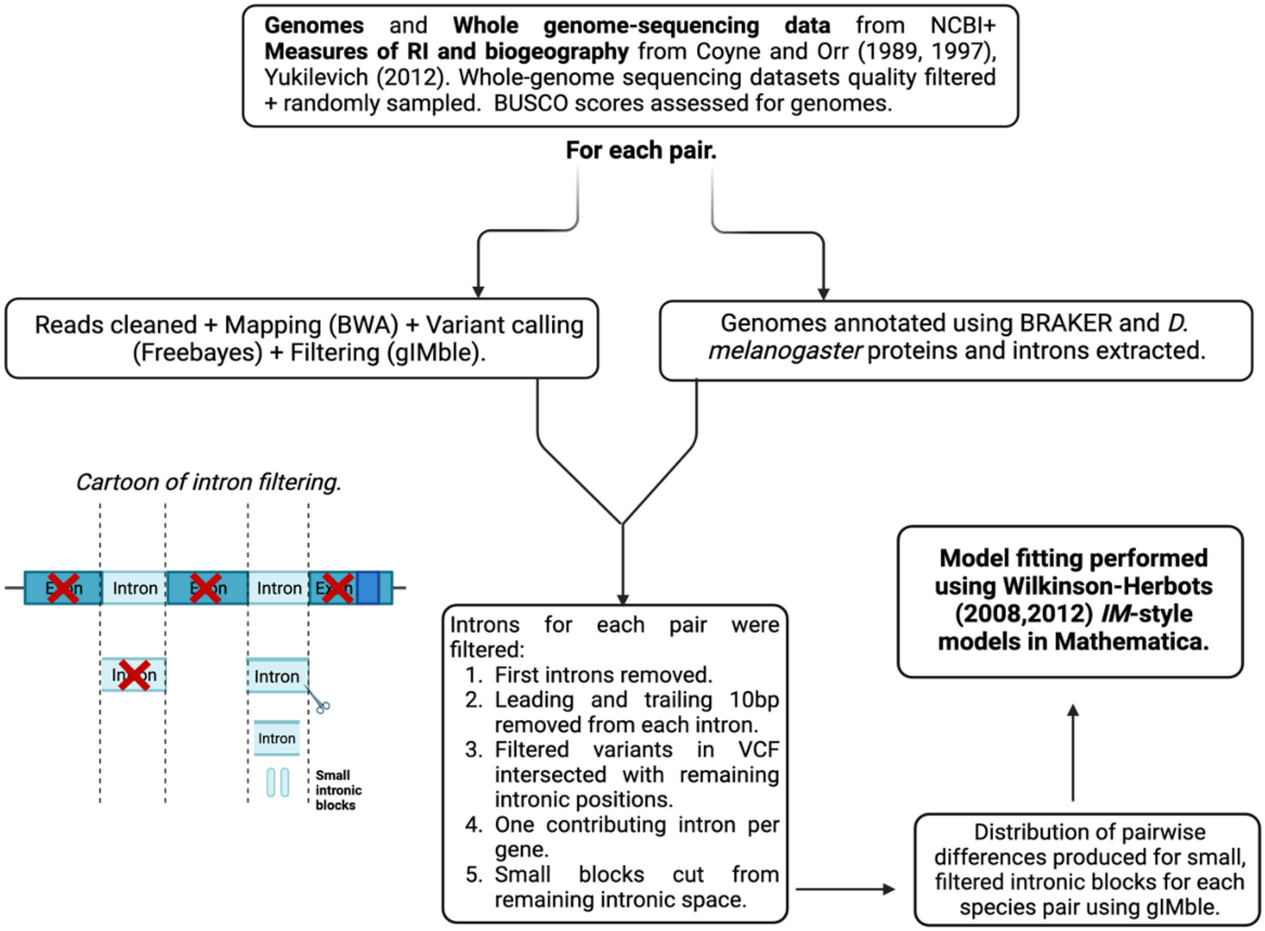

Supplementary Figure 9: Flowchart of analysis pipeline. Describing workflow for the analysis beginning with gathering data on reproductive isolation and biogeography for Drosophila pairs from previous comparative analyses, and whole-genome sequencing datasets and genomes from NCBI, and ending with model fitting for remaining pairs. Intron filtering cartoon alongside the description highlights main filtering decisions taken to produce the datasets used for the model fitting.

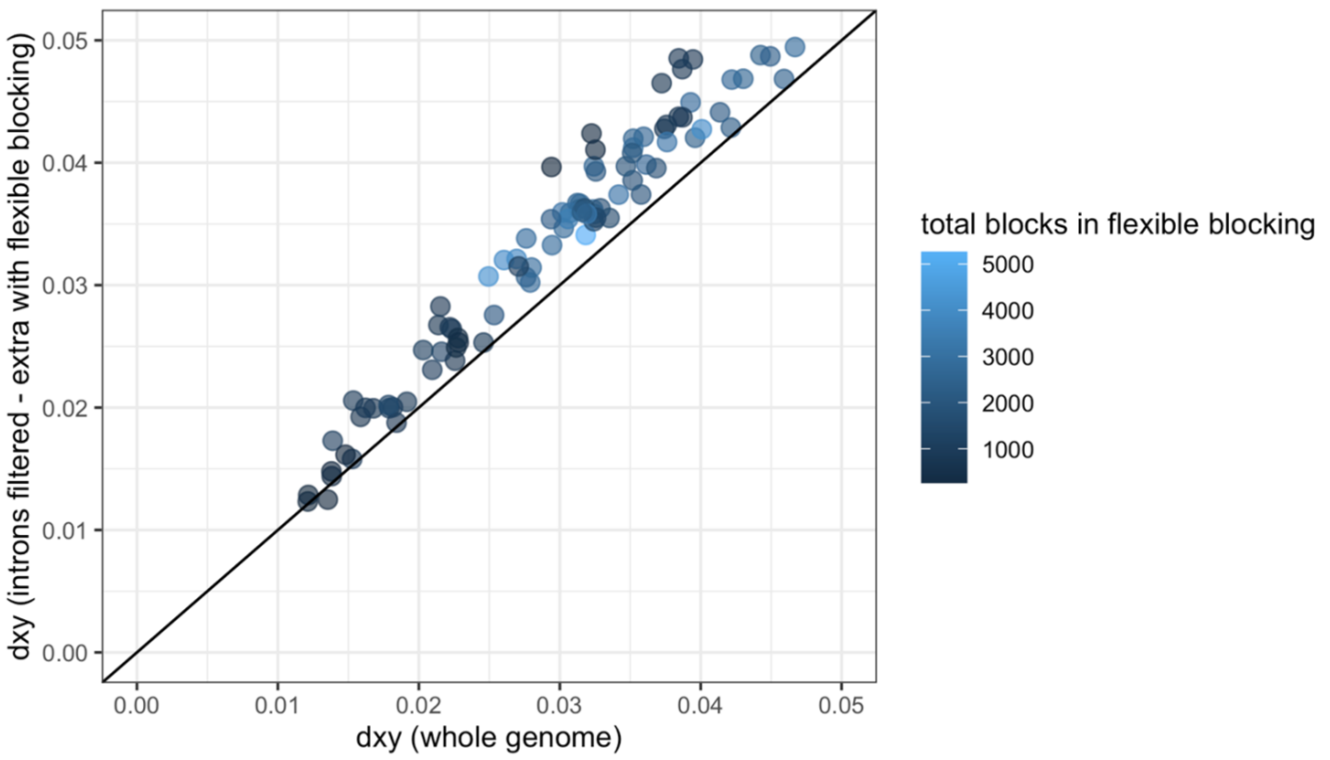

Supplementary Figure 10: The relationship between d_{XY} calculated in fixed 200bp blocks across the whole genome and d_{XY} calculated in filtered, intronic blocks across 93 species pairs. Points are coloured based on how many intronic blocks remained and were used in the model fitting analysis for each pair.

.

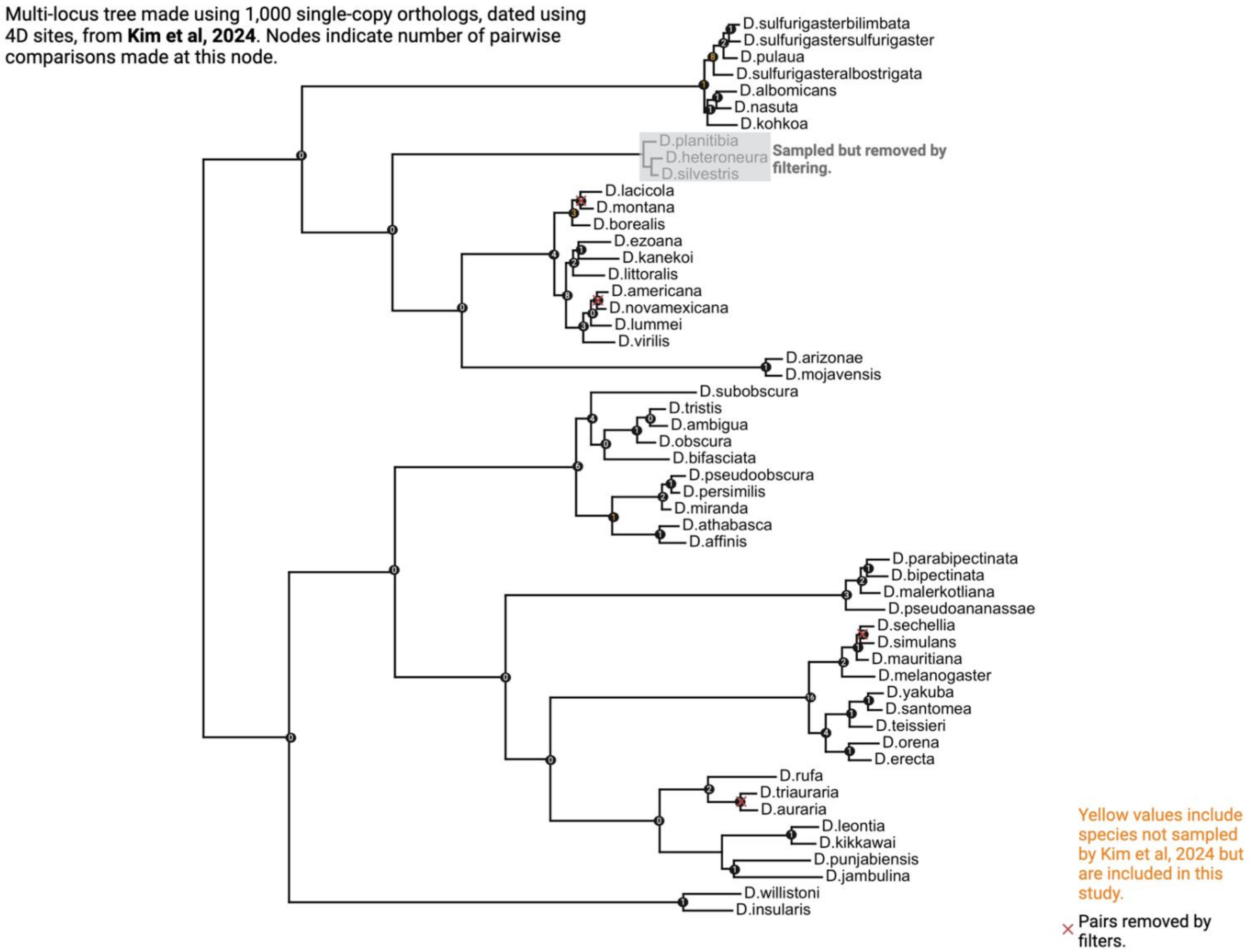

Supplementary Figure 11: Pruned phylogenetic tree of Drosophila taken from Kim et al (2024). Phylogeny contains species considered in this study. Numbers at each node represent the number of pairs that have diverged at these nodes. This is a visualisation representation of the level of non-independence in our dataset.

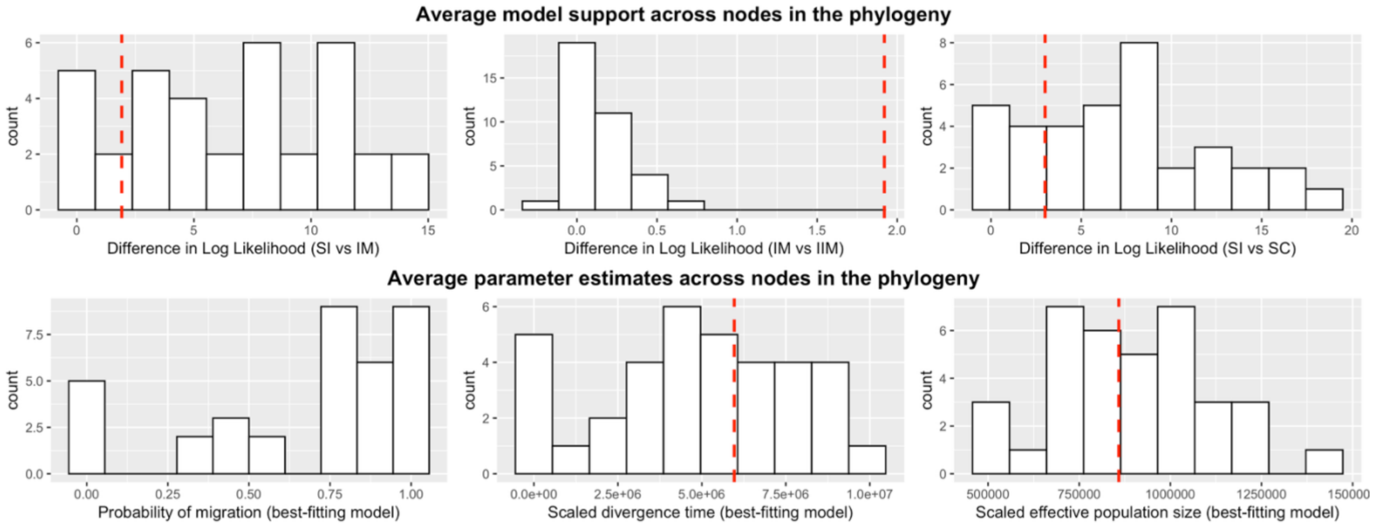

Supplementary Figure 12: Average model support and parameter estimates across nodes in the Drosophila phylogeny. Top panel shows averaged difference in log likelihood across nodes in the phylogeny for nested model comparisons. The red dashed line indicates the critical threshold ($p <$ 0.05). The black dashed line denotes 0, where positive values indicate nodes with average support for an IIM model over the SC model, and vice versa. Bottom panel shows average optimised parameter estimates according to the best-fitting model in pairwise comparisons, across nodes in the phylogeny.

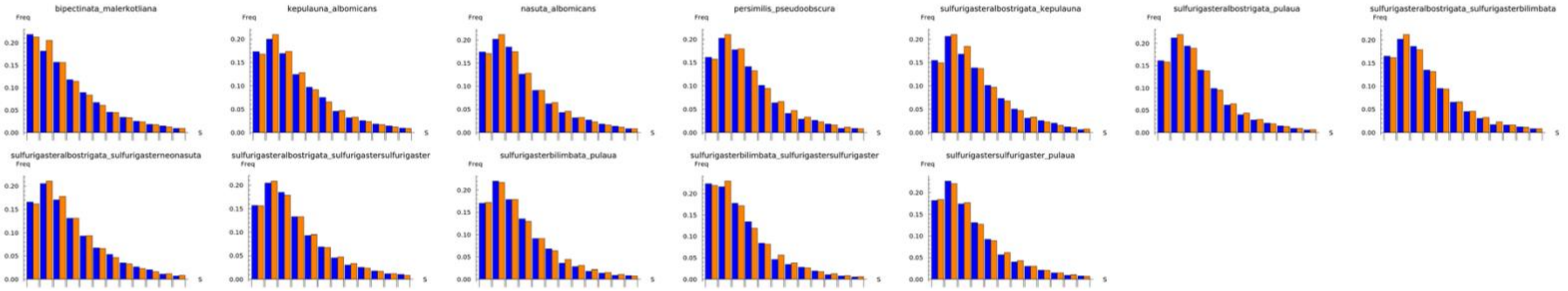

Supplementary Figure 13: Observed (blue) versus expected (orange) S distributions for pairs best fitting a Strict Isolation (SI) model.

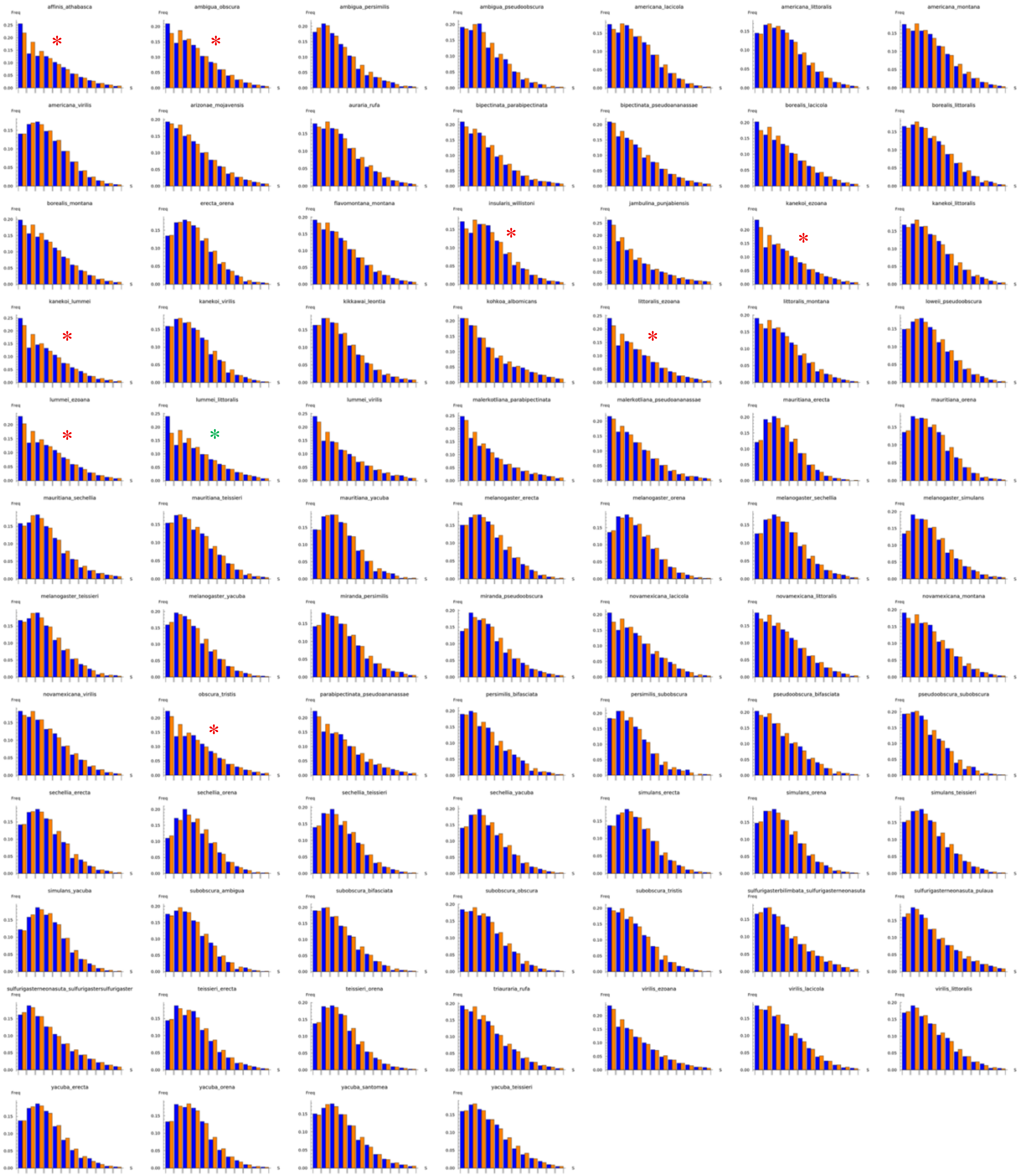

Supplementary Figure 14: Observed (blue) versus expected (orange) S distributions for pairs that fit an IM model significantly better than SI model. Pairs which include red stars best-fit an SC model, and only one pair, with a green star, best-fits the IIM model.

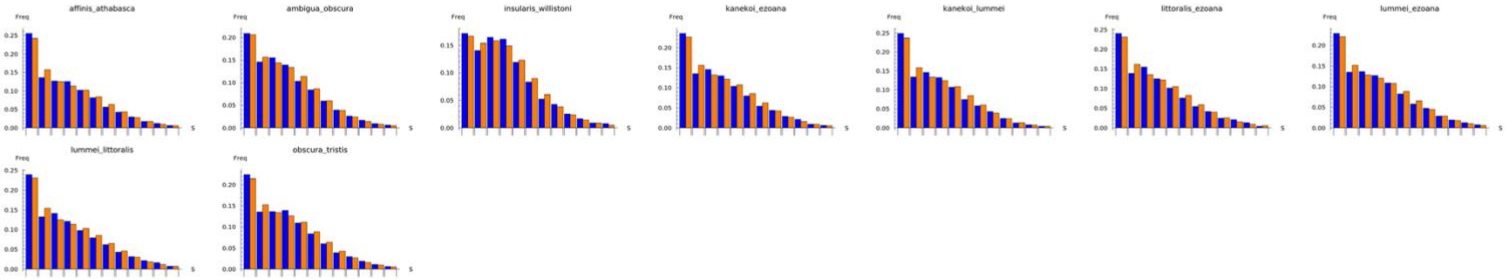

Supplementary Figure 15: Observed (blue) versus expected (orange) S distributions for pairs best fitting a Secondary contact (SC) model

3.2 Tables: 1 - 6

**Pair**	**Accession Species A**	**Accession Species B**
D. affinis - D. athabasca	ERR127384	SRR9967640
D. ambigua - D. obscura	SRR13070667	SRR13070671
D. ambigua - D. persimilis	SRR12899691	SRR13070667
D. ambigua - D. pseudoobscura	SRR11230572	SRR13070667
D. ambigua - D. tristis	SRR13070667	SRR13070669
D. americana - D. lacicola	ERR2610695	SRR20226091
D. americana - D. littoralis	ERR2610695	SRR13070670
D. americana - D. montana	ERR2610695	SRR3218635
D. americana - D. novamexicana	ERR2610692	ERR2610695
D. americana - D. virilis	ERR2610695	SRR9426114
D. arizonae - D. mojavensis	SRR2070760	SRR6425997
D. auraria - D. rufa	SRR13070713	SRR13188937
D. auraria - D. triauraria	SRR13188922	SRR13188937
D. bipectinata - D. malerkotliana (pallens)	SRR13070716	SRR15048573
D. bipectinata - D. parabipectinata	SRR13070721	SRR15048573
D. bipectinata - D. pseudoananassae (nigrens)	SRR13070715	SRR15048573
D. borealis - D. lacicola	SRR20226101	SRR20226091
D. borealis - D. littoralis	SRR13070670	SRR20226101
D. borealis - D. montana	SRR3218635	SRR20226101
D. erecta - D. orena	SRR1977592	SRR6425990
D. flavomontana - D. montana	SRR3218635	SRR20226092
D. heteroneura - D. planitibia	SRR2233094	SRR2233332
D. heteroneura - D. silvestris	SRR2233094	SRR2233314
D. insularis - D. willistoni	SRR13070692	SRR6426003
D. jambulina - D. punjabiensis	SRR15048354	SRR9997923

D. kanekoi - D. ezoana	SRR20226095	SRR20226093
D. kanekoi - D. littoralis	SRR13070670	SRR20226093
D. kanekoi - D. virilis	SRR9426114	SRR20226093
D. kepulauna - D. albomicans	DRR076000	SRR10100373
D. kikkawai - D. leontia	SRR15048447	SRR1810494
D. kohkoa - D. albomicans	DRR076000	DRR160734
D. lacicola - D. montana	SRR3218635	SRR20226091
D. littoralis - D. ezoana	SRR13070670	SRR20226095
D. littoralis - D. montana	SRR13070670	SRR3218635
D. loweii - D. pseudoobscura	SRR11230572	SRR9967663
D. lummei - D. ezoana	SRR5278982	SRR20226095
D. lummei - D. littoralis	SRR13070670	SRR5278982
D. lummei - D. virilis	SRR5278982	SRR9426114
D. malerkotliana (pallens) - D. parabipectinata	SRR13070716	SRR13070721
D. malerkotliana (pallens) - D. pseudoananassae (nigrens)	SRR13070715	SRR13070716
D. mauritiana - D. erecta	SRR1560109	SRR6425990
D. mauritiana - D. orena	SRR1560109	SRR1977592
D. mauritiana - D. sechellia	SRR14138507	SRR1560109
D. mauritiana - D. teissieri	SRR1560109	SRR5860615
D. mauritiana - D. yakuba	SRR1560109	SRR9700025
D. melanogaster - D. erecta	SRR3931586	SRR6425990
D. melanogaster - D. orena	SRR1977592	SRR3931586
D. melanogaster - D. sechellia	SRR14138507	SRR3931586
D. melanogaster - D. simulans	SRR11456787	SRR3931586
D. melanogaster - D. teissieri	SRR3931586	SRR5860615
D. melanogaster - D. yakuba	SRR3931586	SRR9700025

D. miranda - D. bifasciata	DRR061004	SRR789669
D. miranda - D. persimilis	SRR12899691	SRR789669
D. miranda - D. pseudoobscura	SRR11230572	SRR789669
D. miranda - D. subobscura	SRR789669	SRR9967664
D. nasuta - D. albomicans	DRR076000	SRR10100355
D. novamexicana - D. lacicola	ERR2610692	SRR20226091
D. novamexicana - D. littoralis	ERR2610692	SRR13070670
D. novamexicana - D. montana	ERR2610692	SRR3218635
D. novamexicana - D. virilis	ERR2610692	SRR9426114
D. obscura - D. tristis	SRR13070669	SRR13070671
D. parabipectinata - D. pseudoananassae	SRR13070715	SRR13070721
D. persimilis - D. bifasciata	DRR061004	SRR12899691
D. persimilis - D. pseudoobscura	SRR11230572	SRR12899691
D. persimilis - D. subobscura	SRR12899691	SRR9967664
D. planitibia - D. silvestris	SRR2233314	SRR2233332
D. pseudoobscura - D. bifasciata	DRR061004	SRR11230572
D. pseudoobscura - D. subobscura	SRR11230572	SRR9967664
D. sechellia - D. erecta	SRR14138507	SRR6425990
D. sechellia - D. orena	SRR14138507	SRR1977592
D. sechellia - D. teissieri	SRR14138507	SRR5860615
D. sechellia - D. yakuba	SRR14138507	SRR9700025
D. simulans - D. erecta	SRR11456787	SRR6425990
D. simulans - D. orena	SRR11456787	SRR1977592
D. simulans - D. sechellia	SRR11456787	SRR14138507
D. simulans -D. teissieri	SRR11456787	SRR5860615
D. simulans - D. yakuba	SRR11456787	SRR9700025
D. subobscura - D. ambigua	SRR13070667	SRR9967664

D. subobscura - D. bifasciata	DRR061004	SRR9967664
D. subobscura - D. obscura	SRR13070671	SRR9967664
D. subobscura - D. tristis	SRR13070669	SRR9967664
D. sulfurigaster albostrigata - D. kepulauna	SRR10100373	SRR10100407
D. sulfurigaster albostrigata - D. pulaua	SRR10100335	SRR10100407
D. sulfurigaster albostrigata - D. sulfurigaster bilimbata	SRR10100308	SRR10100323
D. sulfurigaster albostrigata - D. sulfurigaster neonasuta	SRR10100407	SRR5059342
D. sulfurigaster albostrigata - D. sulfurigaster sulfurigaster	SRR10100323	SRR10100401
D. sulfurigaster bilimbata - pulaua	SRR10100308	SRR10100335
D. sulfurigaster bilimbata - sulfurigaster neonasuta	SRR10100308	SRR5059342
D. sulfurigaster bilimbata - D. sulfurigaster sulfurigaster	SRR10100308	SRR10100401
D. sulfurigaster neonasuta - pulaua	SRR10100335	SRR5059342
D. sulfurigaster neonasuta - D. sulfurigaster sulfurigaster	SRR10100401	SRR5059342
D. sulfurigaster sulfurigaster - D. pulaua	SRR10100335	SRR10100401
D. teissieri - D. erecta	SRR5860615	SRR6425990
D. teissieri - D. orena	SRR1977592	SRR5860615
D. triauraria - D. rufa	SRR13070713	SRR13188922
D. virilis - D. ezoana	SRR9426114	SRR20226095
D. virilis - D. lacicola	SRR9426114	SRR20226091
D. virilis - D. littoralis	SRR13070670	SRR9426114
D. yakuba - D. erecta	SRR6425990	SRR9700025
D. yakuba - D. orena	SRR1977592	SRR9700025
D. yakuba - D. santomea	SRR5860642	SRR9700025

D. yakuba - D. teissieri	SRR5860615	SRR9700025

Supplementary Table 1: Species pairs and SRA accessions for whole-genome sequencing samples used in this study.

Pair	Intronic D_{XY}	No. of blocks	Block size
D. affinis - D. athabasca	0.028	1300	137
D. ambigua - D. obscura	0.0119	2460	99
D. ambigua - D. persimilis	0.0128	738	70
D. ambigua - D. pseudoobscura	0.0126	725	70
D. americana - D. lacicola	0.0128	2130	82
D. americana - D. littoralis	0.0132	2230	84
D. americana - D. montana	0.0115	2140	82
D. americana - D. virilis	0.0129	2530	84
D. arizonae - D. mojavensis	0.0145	3120	121
D. auraria- D. rufa	0.0132	2080	83
D. bipectinata - D. malerkotliana	0.0108	2670	157
D. bipectinata - D. parabipectinata	0.0116	2660	165
D. bipectinata - D. pseudoananassae	0.0117	1920	116
D. borealis - D. lacicola	0.0133	3270	94
D. borealis - D. littoralis	0.0139	2070	72
D. borealis - D. montana	0.01	3280	93
D. erecta - D. orena	0.0143	2440	95
D. flavomontana- D. montana	0.0103	3460	98
D. insularis - D. willistoni	0.0147	1720	80
D. jambulina - D. punjabiensis	0.0122	2590	109
D. kanekoi - D. ezoana	0.0138	2450	84
D. kanekoi - D. littoralis	0.0144	2160	75
D. kanekoi - D. lummei	0.012	2230	81
D. kanekoi - D. virilis	0.0141	1890	67

D. kepulauna - D. albomicans	0.0125	3510	185
D. kikkawai- D. leontia	0.00865	3010	205
D. kohkoa - D. albomicans	0.0128	3790	111
D. littoralis - D. ezoana	0.0131	2480	89
D. littoralis - D. montana	0.0118	2160	76
D. loweii - D. pseudoobscura	0.0135	2150	98
D. lummei - D. ezoana	0.0125	2450	83
D. lummei - D. littoralis	0.0125	2350	87
D. lummei - D. virilis	0.0107	2680	117
D. malerkotliana - D. parabipectinata	0.0139	2950	159
D. malerkotliana - D. pseudoananassae	0.0123	1950	116
D. mauritiana - D. erecta	0.0138	1580	82
D. mauritiana - D. orena	0.0143	1590	78
D. mauritiana - D. sechellia	0.0148	3140	155
D. mauritiana - D. teissieri	0.0139	1930	72
D. mauritiana - D. yakuba	0.0132	1400	83
D. melanogaster - D. erecta	0.0141	1430	85
D. melanogaster - D. orena	0.0135	3990	73
D. melanogaster - D. sechellia	0.0148	6390	88
D. melanogaster - D. simulans	0.011	2010	118
D. melanogaster - D. teissieri	0.0123	1300	86
D. melanogaster - D. yakuba	0.0126	1180	85
D. miranda - D. persimilis	0.0149	2670	155
D. miranda - D. pseudoobscura	0.0146	2560	146
D. nasuta - D. albomicans	0.0143	3350	218
D. novamexicana - D. lacicola	0.0142	2430	72

D. novamexicana - D. littoralis	0.0144	2560	81
D. novamexicana - D. montana	0.0128	2620	84
D. novamexicana - D. virilis	0.0146	2810	85
D. obscura - D. tristis	0.0123	2530	102
D. parabipectinata - D. pseudoananassae	0.0139	2070	120
D. persimilis - D. bifasciata	0.0133	682	69
D. persimilis - D. pseudoobscura	0.0143	2700	228
D. persimilis - D. subobscura	0.0124	377	72
D. pseudoobscura - D. bifasciata	0.0131	703	68
D. pseudoobscura - D. subobscura	0.0123	367	76
D. sechellia - D. erecta	0.0144	1800	60
D. sechellia - D. orena	0.0147	4900	70
D. sechellia - D. teissieri	0.0133	1660	61
D. sechellia - D. yakuba	0.0139	1600	61
D. simulans - D. erecta	0.0134	1680	64
D. simulans - D. orena	0.0123	1470	75
D. simulans - D. teissieri	0.0119	1710	68
D. simulans - D. yakuba	0.013	1730	70
D. subobscura - D. ambigua	0.0122	673	63
D. subobscura - D. bifasciata	0.0125	669	62
D. subobscura - D. obscura	0.0124	658	64
D. subobscura - D. tristis	0.0118	643	62
D. sulfurigaster albostrigata - D. kepulauna	0.0106	2670	188
D. sulfurigaster albostrigata - D. pulaua	0.0102	1460	291
D. sulfurigaster albostrigata - D. sulfurigaster bilimbata	0.0125	2980	212

D. sulfurigaster albostrigata - D. sulfurigaster neonasuta	0.0111	2920	186
D. sulfurigaster albostrigata - D. sulfurigaster sulfurigaster	0.0126	2950	217
D. sulfurigaster bilimbata - D. pulaua	0.0131	1750	314
D. sulfurigaster bilimbata - D. sulfurigaster neonasuta	0.0137	3300	120
D. sulfurigaster bilimbata - D. sulfurigaster sulfurigaster	0.0117	1710	341
D. sulfurigaster neonasuta - D. pulaua	0.0137	3340	121
D. sulfurigaster neonasuta - D. sulfurigaster sulfurigaster	0.0138	3400	122
D. sulfurigaster sulfurigaster - D. pulaua	0.0125	1620	325
D. teissieri- D. erecta	0.0134	1850	64
D. teissieri - D. orena	0.0125	1640	75
D. triauraria - D. rufa	0.0126	1830	83
D. virilis - D. ezoana	0.0133	1930	85
D. virilis - D. lacicola	0.0142	1800	77
D. virilis - D. littoralis	0.014	1920	72
D. yakuba - D. erecta	0.014	1800	64
D. yakuba - D. orena	0.0136	1590	74
D. yakuba - D. santomea	0.0118	3050	147
D. yakuba - D. teissieri	0.0125	2440	90

Supplementary Table 2: Species pairs and summary statistics calculated from filtered intronic data. Includes estimates of intronic absolute genetic divergence (D_{XY}) for each sample, and the number and size of filtered intronic blocks used in the analysis.

Pair	*Species (Genome)*	Source	Reference
D. affinis - D. athabasca	*D. athabasca*	SAMN12214156	Bracewell, R., Chatla, K., Nalley, M.J. and Bachtrog, D., 2019. Dynamic turnover of centromeres drives karyotype evolution in Drosophila. *Elife*, *8*, p.e49002.
D. ambigua - D. obscura	*D. obscura*	SAMN16729671	Kim, B.Y., Wang, J.R., Miller, D.E., Barmina, O., Delaney, E., Thompson, A., Comeault, A.A., Peede, D., D'Agostino, E.R., Pelaez, J. and Aguilar, J.M., 2021. Highly contiguous assemblies of 101 drosophilid genomes. *Elife*, *10*, p.e66405.
D. ambigua - D. persimilis	*D. persimilis*	SAMN16729701	Kim, B.Y., Wang, J.R., Miller, D.E., Barmina, O., Delaney, E., Thompson, A., Comeault, A.A., Peede, D., D'Agostino, E.R., Pelaez, J. and Aguilar, J.M., 2021. Highly contiguous assemblies of 101 drosophilid genomes. *Elife*, *10*, p.e66405.
D. ambigua - D. pseudoobscura	*D.pseudoobscura*	SAMN16729702	Kim, B.Y., Wang, J.R., Miller, D.E., Barmina, O., Delaney, E., Thompson, A., Comeault, A.A., Peede, D., D'Agostino, E.R., Pelaez, J. and Aguilar, J.M., 2021. Highly contiguous assemblies of 101 drosophilid genomes. *Elife*, *10*, p.e66405.
D. americana - D. lacicola	*D. lacicola*	SAMN29503225	Yusuf, L.H., Tyukmaeva, V., Hoikkala, A. and Ritchie, M.G., 2022. Divergence and introgression among the virilis group of Drosophila. *Evolution Letters*, *6*(6), pp.537-551.

D. americana - D. littoralis	*D. littoralis*	SAMN29503227	Yusuf, L.H., Tyukmaeva, V., Hoikkala, A. and Ritchie, M.G., 2022. Divergence and introgression among the virilis group of Drosophila. *Evolution Letters*, *6*(6), pp.537-551.
D. americana - D. montana	*D. montana*	SAMN29503231	Yusuf, L.H., Tyukmaeva, V., Hoikkala, A. and Ritchie, M.G., 2022. Divergence and introgression among the virilis group of Drosophila. *Evolution Letters*, *6*(6), pp.537-551.
D. americana - D. virilis	*D. virilis*	SAMN16729705	Kim, B.Y., Wang, J.R., Miller, D.E., Barmina, O., Delaney, E., Thompson, A., Comeault, A.A., Peede, D., D'Agostino, E.R., Pelaez, J. and Aguilar, J.M., 2021. Highly contiguous assemblies of 101 drosophilid genomes. *Elife*, *10*, p.e66405.
D. arizonae - D. mojavensis	*D. mojavaensis*	SAMN16729700	Kim, B.Y., Wang, J.R., Miller, D.E., Barmina, O., Delaney, E., Thompson, A., Comeault, A.A., Peede, D., D'Agostino, E.R., Pelaez, J. and Aguilar, J.M., 2021. Highly contiguous assemblies of 101 drosophilid genomes. *Elife*, *10*, p.e66405.
D. auraria- D. rufa	*D. auraria*	SAMN12263403	Bronski, M.J., Martinez, C.C., Weld, H.A. and Eisen, M.B., 2020. Whole genome sequences of 23 species from the Drosophila montium species group (Diptera: Drosophilidae): a resource for testing evolutionary hypotheses. *G3: Genes, Genomes, Genetics*, *10*(5), pp.1443-1455.
D. bipectinata - D. malerkotliana	*D. bipectinata*	SAMN16729609	Kim, B.Y., Wang, J.R., Miller, D.E., Barmina, O., Delaney, E., Thompson, A., Comeault, A.A., Peede, D., D'Agostino, E.R., Pelaez, J. and Aguilar, J.M., 2021. Highly contiguous assemblies of 101 drosophilid genomes. *Elife*, *10*, p.e66405.
D. bipectinata - D. parabipectinata	*D. bipectinata*	SAMN16729609	Kim, B.Y., Wang, J.R., Miller, D.E., Barmina, O., Delaney, E., Thompson, A., Comeault, A.A., Peede, D., D'Agostino, E.R., Pelaez, J. and Aguilar, J.M., 2021. Highly contiguous assemblies of 101 drosophilid genomes. *Elife*, *10*, p.e66405.

D. bipectinata - D. pseudoananassae	*D. bipectinata*	SAMN16729609	Kim, B.Y., Wang, J.R., Miller, D.E., Barmina, O., Delaney, E., Thompson, A., Comeault, A.A., Peede, D., D'Agostino, E.R., Pelaez, J. and Aguilar, J.M., 2021. Highly contiguous assemblies of 101 drosophilid genomes. *Elife*, *10*, p.e66405.
D. borealis - D. lacicola	*D. lacicola*	SAMN29503225	Yusuf, L.H., Tyukmaeva, V., Hoikkala, A. and Ritchie, M.G., 2022. Divergence and introgression among the virilis group of Drosophila. *Evolution Letters*, *6*(6), pp.537-551.
D. borealis - D. littoralis	*D. littoralis*	SAMN29503227	Yusuf, L.H., Tyukmaeva, V., Hoikkala, A. and Ritchie, M.G., 2022. Divergence and introgression among the virilis group of Drosophila. *Evolution Letters*, *6*(6), pp.537-551.
D. borealis - D. montana	*D. montana*	SAMN29503231	Yusuf, L.H., Tyukmaeva, V., Hoikkala, A. and Ritchie, M.G., 2022. Divergence and introgression among the virilis group of Drosophila. *Evolution Letters*, *6*(6), pp.537-551.
D. erecta - D. orena	*D. erecta*	SAMN16729697	Kim, B.Y., Wang, J.R., Miller, D.E., Barmina, O., Delaney, E., Thompson, A., Comeault, A.A., Peede, D., D'Agostino, E.R., Pelaez, J. and Aguilar, J.M., 2021. Highly contiguous assemblies of 101 drosophilid genomes. *Elife*, *10*, p.e66405.
D. flavomontana - D. montana	*D. montana*	SAMN29503231	Yusuf, L.H., Tyukmaeva, V., Hoikkala, A. and Ritchie, M.G., 2022. Divergence and introgression among the virilis group of Drosophila. *Evolution Letters*, *6*(6), pp.537-551.
D. insularis - D. willistoni	*D. willistoni*	SAMN16729706	Kim, B.Y., Wang, J.R., Miller, D.E., Barmina, O., Delaney, E., Thompson, A., Comeault, A.A., Peede, D., D'Agostino, E.R., Pelaez, J. and Aguilar, J.M., 2021. Highly contiguous assemblies of 101 drosophilid genomes. *Elife*, *10*, p.e66405.

D. jambulina - D. punjabiensis	*D. punjabiensis*	SAMN12263408	Bronski, M.J., Martinez, C.C., Weld, H.A. and Eisen, M.B., 2020. Whole genome sequences of 23 species from the Drosophila montium species group (Diptera: Drosophilidae): a resource for testing evolutionary hypotheses. *G3: Genes, Genomes, Genetics*, *10*(5), pp.1443-1455.
D. kanekoi - D. ezoana	*D. ezoana*	SAMN29503221	Yusuf, L.H., Tyukmaeva, V., Hoikkala, A. and Ritchie, M.G., 2022. Divergence and introgression among the virilis group of Drosophila. *Evolution Letters*, *6*(6), pp.537-551.
D. kanekoi - D. littoralis	*D. littoralis*	SAMN29503227	Yusuf, L.H., Tyukmaeva, V., Hoikkala, A. and Ritchie, M.G., 2022. Divergence and introgression among the virilis group of Drosophila. *Evolution Letters*, *6*(6), pp.537-551.
D. kanekoi - D. lummei	*D. lummei*	SAMN29503229	Yusuf, L.H., Tyukmaeva, V., Hoikkala, A. and Ritchie, M.G., 2022. Divergence and introgression among the virilis group of Drosophila. *Evolution Letters*, *6*(6), pp.537-551.
D. kanekoi - D. virilis	*D. virilis*	SAMN16729705	Kim, B.Y., Wang, J.R., Miller, D.E., Barmina, O., Delaney, E., Thompson, A., Comeault, A.A., Peede, D., D'Agostino, E.R., Pelaez, J. and Aguilar, J.M., 2021. Highly contiguous assemblies of 101 drosophilid genomes. *Elife*, *10*, p.e66405.
D. kepulauna - D. albomicans	*D. albomicans*	SAMN12703415 (legacy)	Mai, D., Nalley, M.J. and Bachtrog, D., 2020. Patterns of genomic differentiation in the Drosophila nasuta species complex. *Molecular biology and evolution*, *37*(1), pp.208-220; Wei, K.H.C., Mai, D., Chatla, K. and Bachtrog, D., 2022. Dynamics and impacts of transposable element proliferation in the Drosophila nasuta species group radiation. Molecular Biology and Evolution, 39(5), p.msac080.

D. kikkawai-D. leontia	*D. kikkawai*	SAMN16729629	Kim, B.Y., Wang, J.R., Miller, D.E., Barmina, O., Delaney, E., Thompson, A., Comeault, A.A., Peede, D., D'Agostino, E.R., Pelaez, J. and Aguilar, J.M., 2021. Highly contiguous assemblies of 101 drosophilid genomes. *Elife*, *10*, p.e66405; Suvorov, A., Kim, B.Y., Wang, J., Armstrong, E.E., Peede, D., D'agostino, E.R., Price, D.K., Waddell, P.J., Lang, M., Courtier-Orgogozo, V. and David, J.R., 2022. Widespread introgression across a phylogeny of 155 Drosophila genomes. Current Biology, 32(1), pp.111-123.
D. kohkoa - D. albomicans	*D. albomicans*	SAMN12703415 (legacy)	Mai, D., Nalley, M.J. and Bachtrog, D., 2020. Patterns of genomic differentiation in the Drosophila nasuta species complex. *Molecular biology and evolution*, *37*(1), pp.208-220; Wei, K.H.C., Mai, D., Chatla, K. and Bachtrog, D., 2022. Dynamics and impacts of transposable element proliferation in the Drosophila nasuta species group radiation. Molecular Biology and Evolution, 39(5), p.msac080.
D. littoralis - D. ezoana	*D. ezoana*	SAMN29503221	Yusuf, L.H., Tyukmaeva, V., Hoikkala, A. and Ritchie, M.G., 2022. Divergence and introgression among the virilis group of Drosophila. *Evolution Letters*, *6*(6), pp.537-551.
D. littoralis - D. montana	*D. montana*	SAMN29503231	Yusuf, L.H., Tyukmaeva, V., Hoikkala, A. and Ritchie, M.G., 2022. Divergence and introgression among the virilis group of Drosophila. *Evolution Letters*, *6*(6), pp.537-551.
D. loweii - D. pseudoobscura	*D. pseudoobscura*	SAMN16729702	Kim, B.Y., Wang, J.R., Miller, D.E., Barmina, O., Delaney, E., Thompson, A., Comeault, A.A., Peede, D., D'Agostino, E.R., Pelaez, J. and Aguilar, J.M., 2021. Highly contiguous assemblies of 101 drosophilid genomes. *Elife*, *10*, p.e66405.

D. lummei - D. ezoana	*D. lummei*	SAMN29503229	Yusuf, L.H., Tyukmaeva, V., Hoikkala, A. and Ritchie, M.G., 2022. Divergence and introgression among the virilis group of Drosophila. *Evolution Letters*, *6*(6), pp.537-551.
D. lummei - D. littoralis	*D. lummei*	SAMN29503229	Yusuf, L.H., Tyukmaeva, V., Hoikkala, A. and Ritchie, M.G., 2022. Divergence and introgression among the virilis group of Drosophila. *Evolution Letters*, *6*(6), pp.537-551.
D. lummei - D. virilis	*D. lummei*	SAMN29503229	Yusuf, L.H., Tyukmaeva, V., Hoikkala, A. and Ritchie, M.G., 2022. Divergence and introgression among the virilis group of Drosophila. *Evolution Letters*, *6*(6), pp.537-551.
D. malerkotliana - D. parabipectinata	*D. parabipectinata*	SAMN16729611	Kim, B.Y., Wang, J.R., Miller, D.E., Barmina, O., Delaney, E., Thompson, A., Comeault, A.A., Peede, D., D'Agostino, E.R., Pelaez, J. and Aguilar, J.M., 2021. Highly contiguous assemblies of 101 drosophilid genomes. *Elife*, *10*, p.e66405.
D. malerkotliana - D. pseudoananassae	*D. malerkotliana*	SAMN16729617	Kim, B.Y., Wang, J.R., Miller, D.E., Barmina, O., Delaney, E., Thompson, A., Comeault, A.A., Peede, D., D'Agostino, E.R., Pelaez, J. and Aguilar, J.M., 2021. Highly contiguous assemblies of 101 drosophilid genomes. *Elife*, *10*, p.e66405.
D. mauritiana - D. erecta	*D. erecta*	SAMN16729697	Kim, B.Y., Wang, J.R., Miller, D.E., Barmina, O., Delaney, E., Thompson, A., Comeault, A.A., Peede, D., D'Agostino, E.R., Pelaez, J. and Aguilar, J.M., 2021. Highly contiguous assemblies of 101 drosophilid genomes. *Elife*, *10*, p.e66405.
D. mauritiana - D. orena	*D. mauritiana*	SAMN06827991	No specific publication; see https://www.ncbi.nlm.nih.gov/datasets/genome/GCA_004382145.1/
D. mauritiana - D. sechellia	*D. mauritiana*	SAMN06827991	No specific publication; see https://www.ncbi.nlm.nih.gov/datasets/genome/GCA_004382145.1/

D. mauritiana - D. teissieri	*D. mauritiana*	SAMN06827991	No specific publication; see https://www.ncbi.nlm.nih.gov/datasets/genome/GCA_004382145.1/
D. mauritiana - D. yakuba	*D. yakuba*	SAMN16729707	Kim, B.Y., Wang, J.R., Miller, D.E., Barmina, O., Delaney, E., Thompson, A., Comeault, A.A., Peede, D., D'Agostino, E.R., Pelaez, J. and Aguilar, J.M., 2021. Highly contiguous assemblies of 101 drosophilid genomes. *Elife*, *10*, p.e66405.
D. melanogaster - D. erecta	*D. erecta*	SAMN16729697	Kim, B.Y., Wang, J.R., Miller, D.E., Barmina, O., Delaney, E., Thompson, A., Comeault, A.A., Peede, D., D'Agostino, E.R., Pelaez, J. and Aguilar, J.M., 2021. Highly contiguous assemblies of 101 drosophilid genomes. *Elife*, *10*, p.e66405.
D. melanogaster - D. orena	*D. melanogaster*	dmel_r6.39_FB2021_02/	Flybase; v6.39.
D. melanogaster - D. sechellia	*D. melanogaster*	dmel_r6.39_FB2021_02/	Flybase; v6.39.
D. melanogaster - D. simulans	*D. simulans*	SAMN16729704	Kim, B.Y., Wang, J.R., Miller, D.E., Barmina, O., Delaney, E., Thompson, A., Comeault, A.A., Peede, D., D'Agostino, E.R., Pelaez, J. and Aguilar, J.M., 2021. Highly contiguous assemblies of 101 drosophilid genomes. *Elife*, *10*, p.e66405.
D. melanogaster - D. teissieri	*D. teissieri*	SAMN16729662	Kim, B.Y., Wang, J.R., Miller, D.E., Barmina, O., Delaney, E., Thompson, A., Comeault, A.A., Peede, D., D'Agostino, E.R., Pelaez, J. and Aguilar, J.M., 2021. Highly contiguous assemblies of 101 drosophilid genomes. *Elife*, *10*, p.e66405; Suvorov, A., Kim, B.Y., Wang, J., Armstrong, E.E., Peede, D., D'agostino, E.R., Price, D.K., Waddell, P.J., Lang, M., Courtier-Orgogozo, V. and David, J.R., 2022. Widespread introgression across a phylogeny of 155 Drosophila genomes. Current Biology, 32(1), pp.111-123.

D. melanogaster - D. yakuba	*D. yakuba*	SAMN16729707	Kim, B.Y., Wang, J.R., Miller, D.E., Barmina, O., Delaney, E., Thompson, A., Comeault, A.A., Peede, D., D'Agostino, E.R., Pelaez, J. and Aguilar, J.M., 2021. Highly contiguous assemblies of 101 drosophilid genomes. *Elife*, *10*, p.e66405.
D. miranda - D. persimilis	*D. persimilis*	SAMN16729701	Kim, B.Y., Wang, J.R., Miller, D.E., Barmina, O., Delaney, E., Thompson, A., Comeault, A.A., Peede, D., D'Agostino, E.R., Pelaez, J. and Aguilar, J.M., 2021. Highly contiguous assemblies of 101 drosophilid genomes. *Elife*, *10*, p.e66405.
D. miranda - D. pseudoobscura	*D.pseudoobscura*	SAMN16729702	Kim, B.Y., Wang, J.R., Miller, D.E., Barmina, O., Delaney, E., Thompson, A., Comeault, A.A., Peede, D., D'Agostino, E.R., Pelaez, J. and Aguilar, J.M., 2021. Highly contiguous assemblies of 101 drosophilid genomes. *Elife*, *10*, p.e66405.
D. nasuta - D. albomicans	*D. albomicans*	SAMN12703415 (legacy)	Mai, D., Nalley, M.J. and Bachtrog, D., 2020. Patterns of genomic differentiation in the Drosophila nasuta species complex. *Molecular biology and evolution*, *37*(1), pp.208-220; Wei, K.H.C., Mai, D., Chatla, K. and Bachtrog, D., 2022. Dynamics and impacts of transposable element proliferation in the Drosophila nasuta species group radiation. Molecular Biology and Evolution, 39(5), p.msac080.
D. novamexicana - D. lacicola	*D. novamexicana*	SAMN09383009	No specific publication; see https://www.ncbi.nlm.nih.gov/datasets/genome/GCF_003285875.2/
D. novamexicana - D. littoralis	*D. novamexicana*	SAMN09383009	No specific publication; see https://www.ncbi.nlm.nih.gov/datasets/genome/GCF_003285875.2/
D. novamexicana - D. montana	*D. novamexicana*	SAMN09383009	No specific publication; see https://www.ncbi.nlm.nih.gov/datasets/genome/GCF_003285875.2/

D. novamexicana - D. virilis	*D. novamexicana*	SAMN09383009	No specific publication; see https://www.ncbi.nlm.nih.gov/datasets/genome/GCF_003285875.2/
D. obscura - D. tristis	*D. obscura*	SAMN16729671	Kim, B.Y., Wang, J.R., Miller, D.E., Barmina, O., Delaney, E., Thompson, A., Comeault, A.A., Peede, D., D'Agostino, E.R., Pelaez, J. and Aguilar, J.M., 2021. Highly contiguous assemblies of 101 drosophilid genomes. *Elife*, *10*, p.e66405.
D. parabipectinata - D. pseudoananassae	*D. parabipectinata*	SAMN16729611	Kim, B.Y., Wang, J.R., Miller, D.E., Barmina, O., Delaney, E., Thompson, A., Comeault, A.A., Peede, D., D'Agostino, E.R., Pelaez, J. and Aguilar, J.M., 2021. Highly contiguous assemblies of 101 drosophilid genomes. *Elife*, *10*, p.e66405.
D. persimilis - D. bifasciata	*D. persimilis*	SAMN16729701	Kim, B.Y., Wang, J.R., Miller, D.E., Barmina, O., Delaney, E., Thompson, A., Comeault, A.A., Peede, D., D'Agostino, E.R., Pelaez, J. and Aguilar, J.M., 2021. Highly contiguous assemblies of 101 drosophilid genomes. *Elife*, *10*, p.e66405.
D. persimilis - D. pseudoobscura	*D. persimilis*	SAMN16729701	Kim, B.Y., Wang, J.R., Miller, D.E., Barmina, O., Delaney, E., Thompson, A., Comeault, A.A., Peede, D., D'Agostino, E.R., Pelaez, J. and Aguilar, J.M., 2021. Highly contiguous assemblies of 101 drosophilid genomes. *Elife*, *10*, p.e66405.
D. persimilis - D. subobscura	*D. persimilis*	SAMN16729701	Kim, B.Y., Wang, J.R., Miller, D.E., Barmina, O., Delaney, E., Thompson, A., Comeault, A.A., Peede, D., D'Agostino, E.R., Pelaez, J. and Aguilar, J.M., 2021. Highly contiguous assemblies of 101 drosophilid genomes. *Elife*, *10*, p.e66405.
D. pseudoobscura - D. bifasciata	*D.pseudoobscura*	SAMN16729702	Kim, B.Y., Wang, J.R., Miller, D.E., Barmina, O., Delaney, E., Thompson, A., Comeault, A.A., Peede, D., D'Agostino, E.R., Pelaez, J. and Aguilar, J.M., 2021. Highly contiguous assemblies of 101 drosophilid genomes. *Elife*, *10*, p.e66405.

D. pseudoobscura - D. subobscura	*D.pseudoobscura*	SAMN16729702	Kim, B.Y., Wang, J.R., Miller, D.E., Barmina, O., Delaney, E., Thompson, A., Comeault, A.A., Peede, D., D'Agostino, E.R., Pelaez, J. and Aguilar, J.M., 2021. Highly contiguous assemblies of 101 drosophilid genomes. *Elife*, *10*, p.e66405.
D. sechellia - D. erecta	*D. erecta*	SAMN16729697	Kim, B.Y., Wang, J.R., Miller, D.E., Barmina, O., Delaney, E., Thompson, A., Comeault, A.A., Peede, D., D'Agostino, E.R., Pelaez, J. and Aguilar, J.M., 2021. Highly contiguous assemblies of 101 drosophilid genomes. *Elife*, *10*, p.e66405.
D. sechellia - D. orena	*D. melanogaster*	dmel_r6.39_FB2021_02/	Flybase; v6.39.
D. sechellia - D. teissieri	*D. teissieri*	SAMN16729662	Kim, B.Y., Wang, J.R., Miller, D.E., Barmina, O., Delaney, E., Thompson, A., Comeault, A.A., Peede, D., D'Agostino, E.R., Pelaez, J. and Aguilar, J.M., 2021. Highly contiguous assemblies of 101 drosophilid genomes. *Elife*, *10*, p.e66405; Suvorov, A., Kim, B.Y., Wang, J., Armstrong, E.E., Peede, D., D'agostino, E.R., Price, D.K., Waddell, P.J., Lang, M., Courtier-Orgogozo, V. and David, J.R., 2022. Widespread introgression across a phylogeny of 155 Drosophila genomes. Current Biology, 32(1), pp.111-123.
D. sechellia - D. yakuba	*D. yakuba*	SAMN16729707	Kim, B.Y., Wang, J.R., Miller, D.E., Barmina, O., Delaney, E., Thompson, A., Comeault, A.A., Peede, D., D'Agostino, E.R., Pelaez, J. and Aguilar, J.M., 2021. Highly contiguous assemblies of 101 drosophilid genomes. *Elife*, *10*, p.e66405.
D. simulans - D. erecta	*D. simulans*	SAMN16729704	Kim, B.Y., Wang, J.R., Miller, D.E., Barmina, O., Delaney, E., Thompson, A., Comeault, A.A., Peede, D., D'Agostino, E.R., Pelaez, J. and Aguilar, J.M., 2021. Highly contiguous assemblies of 101 drosophilid genomes. *Elife*, *10*, p.e66405.

D. simulans - D. orena	*D. simulans*	SAMN16729704	Kim, B.Y., Wang, J.R., Miller, D.E., Barmina, O., Delaney, E., Thompson, A., Comeault, A.A., Peede, D., D'Agostino, E.R., Pelaez, J. and Aguilar, J.M., 2021. Highly contiguous assemblies of 101 drosophilid genomes. *Elife*, *10*, p.e66405.
D. simulans - D. teissieri	*D. simulans*	SAMN16729704	Kim, B.Y., Wang, J.R., Miller, D.E., Barmina, O., Delaney, E., Thompson, A., Comeault, A.A., Peede, D., D'Agostino, E.R., Pelaez, J. and Aguilar, J.M., 2021. Highly contiguous assemblies of 101 drosophilid genomes. *Elife*, *10*, p.e66405.
D. simulans - D. yakuba	*D. simulans*	SAMN16729704	Kim, B.Y., Wang, J.R., Miller, D.E., Barmina, O., Delaney, E., Thompson, A., Comeault, A.A., Peede, D., D'Agostino, E.R., Pelaez, J. and Aguilar, J.M., 2021. Highly contiguous assemblies of 101 drosophilid genomes. *Elife*, *10*, p.e66405.
D. subobscura - D. ambigua	*D. subobscura*	SAMN16729670	Kim, B.Y., Wang, J.R., Miller, D.E., Barmina, O., Delaney, E., Thompson, A., Comeault, A.A., Peede, D., D'Agostino, E.R., Pelaez, J. and Aguilar, J.M., 2021. Highly contiguous assemblies of 101 drosophilid genomes. *Elife*, *10*, p.e66405.
D. subobscura - D. bifasciata	*D. subobscura*	SAMN16729670	Kim, B.Y., Wang, J.R., Miller, D.E., Barmina, O., Delaney, E., Thompson, A., Comeault, A.A., Peede, D., D'Agostino, E.R., Pelaez, J. and Aguilar, J.M., 2021. Highly contiguous assemblies of 101 drosophilid genomes. *Elife*, *10*, p.e66405.
D. subobscura - D. obscura	*D. subobscura*	SAMN16729670	Kim, B.Y., Wang, J.R., Miller, D.E., Barmina, O., Delaney, E., Thompson, A., Comeault, A.A., Peede, D., D'Agostino, E.R., Pelaez, J. and Aguilar, J.M., 2021. Highly contiguous assemblies of 101 drosophilid genomes. *Elife*, *10*, p.e66405.

D. subobscura - D. tristis	*D. subobscura*	SAMN16729670	Kim, B.Y., Wang, J.R., Miller, D.E., Barmina, O., Delaney, E., Thompson, A., Comeault, A.A., Peede, D., D'Agostino, E.R., Pelaez, J. and Aguilar, J.M., 2021. Highly contiguous assemblies of 101 drosophilid genomes. *Elife*, *10*, p.e66405.
D. sulfurigaster albostrigata - D. kepulauna	*D. neonasuta*	SAMN06052360	see https://www.ncbi.nlm.nih.gov/datasets/genome/GCA_005889595.1/
D. sulfurigaster albostrigata - D. pulaua	*D. neonasuta*	SAMN06052360	see https://www.ncbi.nlm.nih.gov/datasets/genome/GCA_005889595.1/
D. sulfurigaster albostrigata - D. sulfurigaster bilimbata	*D. neonasuta*	SAMN06052360	see https://www.ncbi.nlm.nih.gov/datasets/genome/GCA_005889595.1/
D. sulfurigaster albostrigata - D. sulfurigaster neonasuta	*D. neonasuta*	SAMN06052360	see https://www.ncbi.nlm.nih.gov/datasets/genome/GCA_005889595.1/
D. sulfurigaster albostrigata - D. sulfurigaster sulfurigaster	*D. neonasuta*	SAMN06052360	see https://www.ncbi.nlm.nih.gov/datasets/genome/GCA_005889595.1/
D. sulfurigaster bilimbata - D. pulaua	*D. neonasuta*	SAMN06052360	see https://www.ncbi.nlm.nih.gov/datasets/genome/GCA_005889595.1/

D. sulfurigaster bilimbata - D. sulfurigaster neonasuta	*D. neonasuta*	SAMN06052360	see https://www.ncbi.nlm.nih.gov/datasets/genome/GCA_005889595.1/
D. sulfurigaster bilimbata - D. sulfurigaster sulfurigaster	*D. neonasuta*	SAMN06052360	see https://www.ncbi.nlm.nih.gov/datasets/genome/GCA_005889595.1/
D. sulfurigaster neonasuta - D. pulaua	*D. neonasuta*	SAMN06052360	see https://www.ncbi.nlm.nih.gov/datasets/genome/GCA_005889595.1/
D. sulfurigaster neonasuta - D. sulfurigaster sulfurigaster	*D. neonasuta*	SAMN06052360	see https://www.ncbi.nlm.nih.gov/datasets/genome/GCA_005889595.1/
D. sulfurigaster sulfurigaster - D. pulaua	*D. neonasuta*	SAMN06052360	see https://www.ncbi.nlm.nih.gov/datasets/genome/GCA_005889595.1/
D. teissieri-D. erecta	*D. teissieri*	SAMN16729662	Kim, B.Y., Wang, J.R., Miller, D.E., Barmina, O., Delaney, E., Thompson, A., Comeault, A.A., Peede, D., D'Agostino, E.R., Pelaez, J. and Aguilar, J.M., 2021. Highly contiguous assemblies of 101 drosophilid genomes. *Elife*, *10*, p.e66405; Suvorov, A., Kim, B.Y., Wang, J., Armstrong, E.E., Peede, D., D'agostino, E.R., Price, D.K., Waddell, P.J., Lang, M., Courtier-Orgogozo, V. and David, J.R., 2022. Widespread introgression across a phylogeny of 155 Drosophila genomes. Current Biology, 32(1), pp.111-123.

D. teissieri - D. orena	*D. teissieri*	SAMN16729662	Kim, B.Y., Wang, J.R., Miller, D.E., Barmina, O., Delaney, E., Thompson, A., Comeault, A.A., Peede, D., D'Agostino, E.R., Pelaez, J. and Aguilar, J.M., 2021. Highly contiguous assemblies of 101 drosophilid genomes. *Elife*, *10*, p.e66405; Suvorov, A., Kim, B.Y., Wang, J., Armstrong, E.E., Peede, D., D'agostino, E.R., Price, D.K., Waddell, P.J., Lang, M., Courtier-Orgogozo, V. and David, J.R., 2022. Widespread introgression across a phylogeny of 155 Drosophila genomes. Current Biology, 32(1), pp.111-123.
D. triauraria - D. rufa	*D. triauraria*	SAMN16744625	Kim, B.Y., Wang, J.R., Miller, D.E., Barmina, O., Delaney, E., Thompson, A., Comeault, A.A., Peede, D., D'Agostino, E.R., Pelaez, J. and Aguilar, J.M., 2021. Highly contiguous assemblies of 101 drosophilid genomes. *Elife*, *10*, p.e66405.
D. virilis - D. ezoana	*D. ezoana*	SAMN29503221	Yusuf, L.H., Tyukmaeva, V., Hoikkala, A. and Ritchie, M.G., 2022. Divergence and introgression among the virilis group of Drosophila. *Evolution Letters*, *6*(6), pp.537-551.
D. virilis - D. lacicola	*D. lacicola*	SAMN29503225	Yusuf, L.H., Tyukmaeva, V., Hoikkala, A. and Ritchie, M.G., 2022. Divergence and introgression among the virilis group of Drosophila. *Evolution Letters*, *6*(6), pp.537-551.
D. virilis - D. littoralis	*D. virilis*	SAMN16729705	Kim, B.Y., Wang, J.R., Miller, D.E., Barmina, O., Delaney, E., Thompson, A., Comeault, A.A., Peede, D., D'Agostino, E.R., Pelaez, J. and Aguilar, J.M., 2021. Highly contiguous assemblies of 101 drosophilid genomes. *Elife*, *10*, p.e66405.
D. yakuba - D. erecta	*D. yakuba*	SAMN16729707	Kim, B.Y., Wang, J.R., Miller, D.E., Barmina, O., Delaney, E., Thompson, A., Comeault, A.A., Peede, D., D'Agostino, E.R., Pelaez, J. and Aguilar, J.M., 2021. Highly contiguous assemblies of 101 drosophilid genomes. *Elife*, *10*, p.e66405.

D. yakuba - D. orena	*D. yakuba*	SAMN16729707	Kim, B.Y., Wang, J.R., Miller, D.E., Barmina, O., Delaney, E., Thompson, A., Comeault, A.A., Peede, D., D'Agostino, E.R., Pelaez, J. and Aguilar, J.M., 2021. Highly contiguous assemblies of 101 drosophilid genomes. *Elife*, *10*, p.e66405.
D. yakuba - D. santomea	*D. yakuba*	SAMN16729707	Kim, B.Y., Wang, J.R., Miller, D.E., Barmina, O., Delaney, E., Thompson, A., Comeault, A.A., Peede, D., D'Agostino, E.R., Pelaez, J. and Aguilar, J.M., 2021. Highly contiguous assemblies of 101 drosophilid genomes. *Elife*, *10*, p.e66405.
D. yakuba - D. teissieri	*D. yakuba*	SAMN16729707	Kim, B.Y., Wang, J.R., Miller, D.E., Barmina, O., Delaney, E., Thompson, A., Comeault, A.A., Peede, D., D'Agostino, E.R., Pelaez, J. and Aguilar, J.M., 2021. Highly contiguous assemblies of 101 drosophilid genomes. *Elife*, *10*, p.e66405.

Supplementary Table 3: Species pairs and the genomes used to map whole-genome sequences to. Includes species' genome which was used for analysis, NCBI BioSample identification of the genome used for each pair and the publications where the data sequenced and assembled (where possible).

Pairs	Sample	Coverage (mean)	Coverage (SD)	Coverage (min)	Coverage (max)
D. affinis - D. athabasca	SRR9967640	13.35	7.86	2	29
D. affinis - D. athabasca	ERR127384	16.24	16.86	2	49
D. ambigua - D. obscura	SRR13070667	18.55	15.92	2	50
D. ambigua - D. obscura	SRR13070671	16.3	10.81	2	37
D. ambigua - D. persimilis	SRR12899691	24.45	19.76	2	63
D. ambigua - D. persimilis	SRR13070667	16.89	20.92	2	58
D. ambigua - D. pseudoobscura	SRR11230572	25.82	14.29	2	54
D. ambigua - D. pseudoobscura	SRR13070667	16.93	22.35	2	61
D. ambigua - D. pseudoobscura	SRR13070669	15.01	30.76	2	76
D. ambigua - D. pseudoobscura	SRR13070667	14.68	25.11	2	64
D. americana - D. lacicola	ERR2610695	16.82	44.13	2	105
D. americana - D. lacicola	SRR20226091	22	8.95	2	39
D. americana - D. littoralis	ERR2610695	16.71	22.26	2	61
D. americana - D. littoralis	SRR13070670	16.28	33.6	2	83
D. americana - D. montana	SRR3218635	28.99	56.14	2	141
D. americana - D. montana	ERR2610695	16.66	209.99	2	436
D. americana - D. novamexicana	ERR2610692	19.69	61.5	2	142
D. americana - D. novamexicana	ERR2610695	19.83	48.49	2	116
D. americana - D. virilis	SRR9426114	9.94	8.52	2	26
D. americana - D. virilis	ERR2610695	18.23	47.92	2	114
D. arizonae - D. mojavensis	SRR2070760	10.93	10.28	2	31
D. arizonae - D. mojavensis	SRR6425997	30.01	15.74	2	61
D. auraria - D. rufa	SRR13188937	11.34	10.05	2	31
D. auraria - D. rufa	SRR13070713	14.91	16.77	2	48
D. auraria - D. triauraria	SRR13188937	11.65	9.47	2	30
D. auraria - D. triauraria	SRR13188922	16.05	12.22	2	40

D. bipectinata - D. malerkotliana (pallens)	SRR15048573	9.74	8.91	2	27
D. bipectinata - D. malerkotliana (pallens)	SRR13070716	19.17	14.54	2	48
D. bipectinata - D. parabipectinata	SRR13070721	12.33	12.51	2	37
D. bipectinata - D. parabipectinata	SRR15048573	9.74	8.91	2	27
D. bipectinata - D. pseudoananassae (nigrens)	SRR15048573	9.74	8.91	2	27
D. bipectinata - D. pseudoananassae (nigrens)	SRR13070715	11.19	17.17	2	45
D. borealis - D. lacicola	SRR13070670	21.49	18.57	2	58
D. borealis - D. lacicola	SRR20226091	22	8.95	2	39
D. borealis - D. littoralis	SRR13070670	16.28	33.6	2	83
D. borealis - D. littoralis	SRR13070670	20.35	17.55	2	55
D. borealis - D. montana	SRR3218635	28.99	56.13	2	141
D. borealis - D. montana	SRR13070670	20.76	14.82	2	50
D. erecta - D. orena	SRR1977592	9.8	8.49	2	26
D. erecta - D. orena	SRR6425990	40.14	17.62	2	75
D. flavomontana - D. montana	SRR3218635	28.99	56.15	2	141
D. flavomontana - D. montana	SRR20226092	21.67	87.7	2	197
D. heteroneura - D. planitibia	SRR2233094	17.54	12.91	2	43
D. heteroneura - D. planitibia	SRR2233332	29.52	202.99	2	435
D. heteroneura - D. silvestris	SRR2233094	17.54	12.91	2	43
D. heteroneura - D. silvestris	SRR2233314	18.35	12.57	2	43
D. insularis - D. willistoni	SRR6426003	23.82	19.33	2	62
D. insularis - D. willistoni	SRR13070692	31.88	37.87	2	107
D. jambulina - D. punjabiensis	SRR9997923	18.72	9.44	2	37
D. jambulina - D. punjabiensis	SRR15048354	7	23.94	2	54
D. kanekoi - D. ezoana	SRR20226095	22.75	10.2	2	43

D. kanekoi - D. ezoana	SRR20226093	19.5	461.55	2	942
D. kanekoi - D. littoralis	SRR13070670	16.28	33.6	2	83
D. kanekoi - D. littoralis	SRR20226093	20.22	24.8	2	69
D. kanekoi - D. virilis	SRR20226093	19.6	282.26	2	584
D. kanekoi - D. virilis	SRR5278982	16.68	12.33	2	41
kanekoi_virilis	SRR9426114	9.94	8.52	2	26
kanekoi_virilis	SRR20226093	20.62	12.68	2	45
D. kepulauna - D. albomicans	DRR076000	82.24	454.35	2	990
D. kepulauna - D. albomicans	SRR10100373	9.19	5.66	2	20
D. kikkawai - D. leontia	SRR1810494	9.04	8.68	2	26
D. kikkawai - D. leontia	SRR15048447	9.23	10.13	2	29
D. kohkoa - D. albomicans	DRR160734	49.97	68.1	2	186
D. kohkoa - D. albomicans	DRR076000	82.24	454.36	2	990
D. lacicola - D. montana	SRR3218635	28.99	56.14	2	141
D. lacicola - D. montana	SRR20226091	20.65	10.58	2	41
D. littoralis - D. ezoana	SRR20226095	22.75	10.2	2	43
D. littoralis - D. ezoana	SRR13070670	15.09	58.14	2	131
D. littoralis - D. montana	SRR13070670	15.21	36.84	2	88
D. littoralis - D. montana	SRR3218635	28.99	56.13	2	141
D. loweii - D. pseudoobscura	SRR11230572	25.82	14.29	2	54
D. loweii - D. pseudoobscura	SRR9967663	12.31	18.12	2	48
D. lummei - D. ezoana	SRR20226095	21.72	19.62	2	60
D. lummei - D. ezoana	SRR5278982	16.68	12.33	2	41
D. lummei - D. littoralis	SRR13070670	15.58	34.12	2	83
D. lummei - D. littoralis	SRR5278982	16.68	12.33	2	41
D. lummei - D. virilis	SRR9426114	8.86	8.21	2	25
D. lummei - D. virilis	SRR5278982	16.68	12.33	2	41
D. malerkotliana (pallens) - D. parabipectinata	SRR13070721	11.7	8.39	2	28

D. malerkotliana (pallens) - D. parabipectinata	SRR13070716	18.9	13.71	2	46
D. malerkotliana (pallens) - D. pseudoananassae (nigrens)	SRR13070716	19.54	11.85	2	43
D. malerkotliana (pallens) - D. pseudoananassae (nigrens)	SRR13070715	11.3	15.78	2	42
D. mauritiana - D. erecta	SRR1560109	12.82	8.14	2	29
D. mauritiana - D. erecta	SRR6425990	40.14	17.62	2	75
D. mauritiana - D. orena	SRR1560109	15.23	7.23	2	29
D. mauritiana - D. orena	SRR1977592	8.85	7.3	2	23
D. mauritiana - D. sechellia	SRR1560109	15.23	7.23	2	29
D. mauritiana - D. sechellia	SRR14138507	22.38	13.35	2	49
D. mauritiana - D. teissieri	SRR5860615	11.89	12.18	2	36
D. mauritiana - D. teissieri	SRR1560109	15.23	7.23	2	29
D. mauritiana - D. yakuba	SRR1560109	12.79	23.88	2	60
D. mauritiana - D. yakuba	SRR9700025	16.48	7.73	2	31
D. melanogaster - D. erecta	SRR3931586	13.19	132.76	2	278
D. melanogaster - D. erecta	SRR6425990	40.14	17.62	2	75
D. melanogaster - D. orena	SRR1977592	8.98	7.93	2	24
D. melanogaster - D. orena	SRR3931586	12.21	12.3	2	36
D. melanogaster - D. sechellia	SRR14138507	21.87	34.25	2	90
D. melanogaster - D. sechellia	SRR3931586	12.21	12.3	2	36
D. melanogaster - D. simulans	SRR11456787	26.86	12.34	2	51
D. melanogaster - D. simulans	SRR3931586	13.15	13.95	2	41
D. melanogaster - D. teissieri	SRR5860615	12.82	7.06	2	26
D. melanogaster - D. teissieri	SRR3931586	12.83	89.37	2	191
D. melanogaster - D. yakuba	SRR9700025	16.48	7.73	2	31
D. melanogaster - D. yakuba	SRR3931586	12.84	78.35	2	169

D. miranda - D. bifasciata	SRR789669	9.27	10.01	2	29
D. miranda - D. bifasciata	DRR061004	46.87	24.65	2	96
D. miranda - D. persimilis	SRR12899691	24.45	19.76	2	63
D. miranda - D. persimilis	SRR789669	13.26	11	2	35
D. miranda - D. pseudoobscura	SRR11230572	25.82	14.29	2	54
D. miranda - D. pseudoobscura	SRR789669	13.23	11.16	2	35
D. miranda - D. subobscura	SRR789669	9.3	8.52	2	26
D. miranda - D. subobscura	SRR9967664	14.94	8.94	2	32
D. nasuta - D. albomicans	SRR10100355	11.68	6.74	2	25
D. nasuta - D. albomicans	DRR076000	82.24	454.36	2	990
D. novamexicana - D. lacicola	SRR20226091	20.59	19.38	2	59
D. novamexicana - D. lacicola	ERR2610692	19.7	61.49	2	142
D. novamexicana - D. littoralis	ERR2610692	19.69	61.48	2	142
D. novamexicana - D. littoralis	SRR13070670	16.35	20.22	2	56
D. novamexicana - D. montana	SRR3218635	25.21	95.57	2	216
D. novamexicana - D. montana	ERR2610692	19.7	61.51	2	142
D. novamexicana - D. virilis	SRR9426114	9.32	8.61	2	26
D. novamexicana - D. virilis	ERR2610692	19.7	61.48	2	142
D. obscura - D. tristis	SRR13070671	16.3	10.81	2	37
D. obscura - D. tristis	SRR13070669	19.21	18.36	2	55
D. parabipectinata - D. pseudoananassae	SRR13070715	10.96	17.22	2	45
D. parabipectinata - D. pseudoananassae	SRR13070721	11.7	8.39	2	28
D. persimilis - D. bifasciata	SRR12899691	24.45	19.76	2	63
D. persimilis - D. bifasciata	DRR061004	37.59	29.98	2	97
D. persimilis - D. pseudoobscura	SRR11230572	25.43	15.99	2	57

D. persimilis - D. pseudoobscura	SRR12899691	24.45	19.76	2	63
D. persimilis - D. subobscura	SRR12899691	24.45	19.76	2	63
D. persimilis - D. subobscura	SRR9967664	11.32	8.56	2	28
D. planitibia - D. silvestris	SRR2233314	18.35	12.57	2	43
D. planitibia - D. silvestris	SRR2233332	29.52	203.01	2	435
D. pseudoobscura - D. bifasciata	SRR11230572	25.82	14.29	2	54
D. pseudoobscura - D. bifasciata	DRR061004	37.7	28.83	2	95
D. pseudoobscura - D. subobscura	SRR11230572	25.82	14.29	2	54
D. pseudoobscura - D. subobscura	SRR9967664	11.35	9.13	2	29
D. sechellia - D. erecta	SRR14138507	21.37	17.45	2	56
D. sechellia - D. erecta	SRR6425990	40.14	17.62	2	75
D. sechellia - D. orena	SRR1977592	8.98	7.93	2	24
D. sechellia - D. orena	SRR14138507	21.87	34.25	2	90
D. sechellia - D. teissieri	SRR5860615	12.82	7.06	2	26
D. sechellia - D. teissieri	SRR14138507	21.06	17.34	2	55
D. sechellia - D. yakuba	SRR14138507	21.14	23.78	2	68
D. sechellia - D. yakuba	SRR9700025	16.48	7.73	2	31
D. simulans - D. erecta	SRR6425990	34.9	19.93	2	74
D. simulans - D. erecta	SRR11456787	26.86	12.34	2	51
D. simulans - D. orena	SRR1977592	9.22	8.4	2	26
D. simulans - D. orena	SRR11456787	26.86	12.34	2	51
D. simulans - D. sechellia	SRR14138507	24.04	15.67	2	55
D. simulans - D. sechellia	SRR11456787	26.86	12.34	2	51
D. simulans - D. teissieri	SRR5860615	12.52	11.5	2	35
D. simulans - D. teissieri	SRR11456787	26.86	12.34	2	51

D. simulans - D. yakuba	SRR11456787	26.86	12.34	2	51
D. simulans - D. yakuba	SRR9700025	12.97	8.79	2	30
D. subobscura - D. ambigua	SRR9967664	14.94	8.94	2	32
D. subobscura - D. ambigua	SRR13070667	16.97	25.77	2	68
D. subobscura - D. bifasciata	SRR9967664	14.94	8.94	2	32
D. subobscura - D. bifasciata	DRR061004	36.99	33.81	2	104
D. subobscura - D. obscura	SRR13070671	14.36	31.75	2	77
D. subobscura - D. obscura	SRR9967664	14.94	8.94	2	32
D. subobscura - D. tristis	SRR9967664	14.94	8.94	2	32
D. subobscura - D. tristis	SRR13070669	17.07	42.16	2	101
D. sulfurigaster albostrigata - D. kepulauna	SRR10100407	5.58	6.02	2	17
D. sulfurigaster albostrigata - D. kepulauna	SRR10100373	9.41	13.34	2	36
D. sulfurigaster albostrigata - D. pulaua	SRR10100407	5.58	6.01	2	17
D. sulfurigaster albostrigata - D. pulaua	SRR10100335	17.39	91.72	2	200
D. sulfurigaster albostrigata - D. sulfurigaster bilimbata	SRR10100323	26.51	21.29	2	69
D. sulfurigaster albostrigata - D. sulfurigaster bilimbata	SRR10100308	15.76	19.18	2	54
D. sulfurigaster albostrigata - D. sulfurigaster neonasuta	SRR5059342	13.07	180.59	2	374
D. sulfurigaster albostrigata - D. sulfurigaster neonasuta	SRR10100407	5.58	6.01	2	17
D. sulfurigaster albostrigata - D. sulfurigaster sulfurigaster	SRR10100323	26.51	21.29	2	69
D. sulfurigaster albostrigata - D.	SRR10100401	14.69	46.96	2	108

sulfurigaster sulfurigaster					
D. sulfurigaster bilimbata - pulaua	SRR10100335	17.39	91.72	2	200
D. sulfurigaster bilimbata - pulaua	SRR10100308	15.76	19.18	2	54
D. sulfurigaster bilimbata - sulfurigaster neonasuta	SRR5059342	13.07	180.59	2	374
D. sulfurigaster bilimbata - sulfurigaster neonasuta	SRR10100308	15.76	19.18	2	54
D. sulfurigaster bilimbata - D. sulfurigaster sulfurigaster	SRR10100308	15.76	19.18	2	54
D. sulfurigaster bilimbata - D. sulfurigaster sulfurigaster	SRR10100401	14.69	46.96	2	108
D. sulfurigaster neonasuta - pulaua	SRR5059342	13.07	180.59	2	374
D. sulfurigaster neonasuta - pulaua	SRR10100335	17.39	91.72	2	200
D. sulfurigaster neonasuta - D. sulfurigaster sulfurigaster	SRR10100401	14.69	46.96	2	108
D. sulfurigaster neonasuta - D. sulfurigaster sulfurigaster	SRR5059342	13.07	180.59	2	374
D. sulfurigaster sulfurigaster - D. pulaua	SRR10100401	14.69	46.96	2	108
D. sulfurigaster sulfurigaster - D. pulaua	SRR10100335	17.39	91.72	2	200
D. teissieri - D. erecta	SRR5860615	12.82	7.06	2	26
D. teissieri - D. erecta	SRR6425990	34.55	20.9	2	76
D. teissieri - D. orena	SRR5860615	12.82	7.06	2	26
D. teissieri - D. orena	SRR1977592	9.24	8.39	2	26
D. triauraria - D. rufa	SRR13188922	16.05	12.22	2	40

D. triauraria - D. rufa	SRR13070713	14.98	16.41	2	47
D. virilis - D. ezoana	SRR20226095	22.75	10.2	2	43
D. virilis - D. ezoana	SRR9426114	8.38	9.33	2	27
D. virilis - D. lacicola	SRR9426114	8.68	8.47	2	25
D. virilis - D. lacicola	SRR20226091	22	8.95	2	39
D. virilis - D. littoralis	SRR9426114	9.94	8.52	2	26
D. virilis - D. littoralis	SRR13070670	16.41	21.28	2	58
D. yakuba - D. erecta	SRR9700025	16.48	7.73	2	31
D. yakuba - D. erecta	SRR6425990	34.64	64.51	2	163
D. yakuba - D. orena	SRR1977592	9.24	8.4	2	26
D. yakuba - D. orena	SRR9700025	16.48	7.73	2	31
D. yakuba - D. santomea	SRR9700025	16.48	7.73	2	31
D. yakuba - D. santomea	SRR5860642	24.27	14.01	2	52
D. yakuba - D. teissieri	SRR5860615	13	9.41	2	31
D. yakuba - D. teissieri	SRR9700025	16.48	7.73	2	31

Supplementary Table 4: Species pairs and sample specific coverage information. Mean, standard deviation, minimum and maximum coverage are provided for each species in a pair-specific manner.

Pair	Premating Isolation (Yukilevich 2014)	Postzygotic Isolation (Yukilevich 2014)	Nei's *D* *(Yukilevich 2014)*	Biogeographic classification (Yukilevich 2014)
D. affinis - D. athabasca	0.775519466	NA	0.74	Sympatry
D. ambigua - D. obscura	0.993894994	NA	0.535	Sympatry
D. ambigua - D. persimilis	NA	1	1.66	Allopatry
D. ambigua - D. pseudoobscura	NA	1	1.66	Allopatry
D. americana - D. lacicola	1	NA	1.42	Sympatry
D. americana - D. littoralis	0.756484913	NA	1.21	Allopatry
D. americana - D. montana	0.992359932	1	1.48	Sympatry
D. americana - D. virilis	0.64428754	0	0.54	Allopatry
D. arizonae - D. mojavensis	0.766970618	0.375	0.279	Allopatry
D. auraria- D. rufa	0.987677141	NA	0.9245	Sympatry
D. bipectinata - D. malerkotliana	0.838798283	0.5	0.104	Sympatry
D. bipectinata - D. parabipectinata	0.55012987	0.5	0.145	Sympatry
D. bipectinata - D. pseudoananassae	0.840869565	NA	0.271	Sympatry
D. borealis - D. lacicola	1	NA	0.34	Sympatry
D. borealis - D. littoralis	1	NA	0.68	Allopatry
D. borealis - D. montana	0.994594595	0.5	0.21	Sympatry
D. erecta - D. orena	1	NA	1.03	Sympatry
D. flavomontana- D. montana	0.992857143	0.5	0.29	Sympatry
D. insularis - D. willistoni	0.923036649	NA	1.07	Sympatry
D. jambulina - D. punjabiensis	0.991326531	NA	0.944	Sympatry
D. kanekoi - D. ezoana	0.9945	NA	1.22	Sympatry
D. kanekoi - D. littoralis	0.5515	NA	1.22	Allopatry
D. kanekoi - D. lummei	0.722052705	NA	1.1	Sympatry
D. kanekoi - D. virilis	0.691660291	NA	1.1	Sympatry
D. kepulauna - D. albomicans	0.939064857	0	0.792	Allopatry
D. kikkawai- D. leontia	0.971875	0	0.244	Sympatry
D. kohkoa - D. albomicans	1	0.75	0.833	Sympatry
D. littoralis - D. ezoana	0.84125	NA	0.65	Sympatry

D. littoralis - D. montana	0.784	0.75	0.66	Sympatry
D. loweii - D. pseudoobscura	0.98	NA	1.05	Sympatry
D. lummei - D. ezoana	1	NA	1.22	Sympatry
D. lummei - D. littoralis	0.629	NA	1.22	Sympatry
D. lummei - D. virilis	0.263	0	0.35	Sympatry
D. malerkotliana - D. parabipectinata	0.853214286	0.625	0.226	Sympatry
D. malerkotliana - D. pseudoananassae	0.956950673	0.95	0.282	Sympatry
D. mauritiana - D. erecta	0.984042553	NA	1.59	Allopatry
D. mauritiana - D. orena	0.9953125	NA	1.07	Allopatry
D. mauritiana - D. sechellia	0.920463693	0.5	0.32	Allopatry
D. mauritiana - D. teissieri	0.875842697	NA	1.24	Allopatry
D. mauritiana - D. yakuba	0.989247312	NA	0.88	Allopatry
D. melanogaster - D. erecta	1	NA	1.63	Sympatry
D. melanogaster - D. orena	1	NA	1.14	Sympatry
D. melanogaster - D. sechellia	0.619439164	1	0.62	Allopatry
D. melanogaster - D. simulans	0.826903313	1	0.55	Sympatry
D. melanogaster - D. teissieri	1	NA	1.01	Sympatry
D. melanogaster - D. yakuba	0.734042553	NA	0.94	Sympatry
D. miranda - D. persimilis	1	1	0.56	Sympatry
D. miranda - D. pseudoobscura	0.99537037	1	0.56	Sympatry
D. nasuta - D. albomicans	0.068400226	0	0.45	Allopatry
D. novamexicana - D. lacicola	1	NA	1.2	Allopatry
D. novamexicana - D. littoralis	0.788918206	NA	0.97	Allopatry
D. novamexicana - D. montana	1	0.75	1.22	Sympatry
D. novamexicana - D. virilis	0.507460036	0	0.5	Allopatry
D. obscura - D. tristis	0.989746683	NA	0.47	Sympatry

D. parabipectinata - D. pseudoananassae	0.762171946	NA	0.366	Sympatry
D. persimilis - D. bifasciata	0.975	NA	1.95	Allopatry
D. persimilis - D. pseudoobscura	0.785042646	0.5	0.41	Sympatry
D. persimilis - D. subobscura	0.99137931	NA	1.66	Allopatry
D. pseudoobscura - D. bifasciata	0.941935484	NA	1.95	Allopatry
D. pseudoobscura - D. subobscura	0.962121212	NA	1.66	Allopatry
D. sechellia - D. erecta	1	NA	1.51	Allopatry
D. sechellia - D. orena	1	NA	1.27	Allopatry
D. sechellia - D. teissieri	0.995	NA	1.36	Allopatry
D. sechellia - D. yakuba	1	NA	1.27	Allopatry
D. simulans - D. erecta	0.997382199	NA	1.5	Sympatry
D. simulans - D. orena	0.953846154	NA	1.01	Sympatry
D. simulans - D. teissieri	0.961325967	NA	1.24	Sympatry
D. simulans - D. yakuba	0.958730159	NA	1	Sympatry
D. subobscura - D. ambigua	0.929569267	NA	1.44	Sympatry
D. subobscura - D. bifasciata	0.934782609	NA	1.33	Allopatry
D. subobscura - D. obscura	1	NA	1.25	Sympatry
D. subobscura - D. tristis	0.98273878	NA	1.1	Sympatry
D. sulfurigaster albostrigata - D. kepulauna	0.979381443	1	0.902	Sympatry
D. sulfurigaster albostrigata - D. pulaua	0.992078001	0	0.389	Sympatry
D. sulfurigaster albostrigata - D. sulfurigaster bilimbata	0.430365297	0.25	0.386	Allopatry
D. sulfurigaster albostrigata - D. sulfurigaster neonasuta	0.183673469	0	0.557	Allopatry
D. sulfurigaster albostrigata - D. sulfurigaster sulfurigaster	0.132816824	0	0.378	Allopatry
D. sulfurigaster bilimbata - D. pulaua	0.921381775	0	0.448	Allopatry

D. sulfurigaster bilimbata - D. sulfurigaster neonasuta	0.595550485	0.5	0.491	Allopatry
D. sulfurigaster bilimbata - D. sulfurigaster sulfurigaster	0.097560976	0	0.555	Allopatry
D. sulfurigaster neonasuta - D. pulaua	0.909866017	0.5	0.705	Allopatry
D. sulfurigaster neonasuta - D. sulfurigaster sulfurigaster	0.415376106	0.5	0.6	Allopatry
D. sulfurigaster sulfurigaster - D. pulaua	0.785467128	0	0.561	Allopatry
D. teissieri- D. erecta	0.977011494	NA	1.54	Sympatry
D. teissieri - D. orena	1	NA	1.47	Sympatry
D. triauraria - D. rufa	0.963319303	NA	0.7335	Sympatry
D. virilis - D. ezoana	0.9645	NA	1.22	Sympatry
D. virilis - D. lacicola	0.799457995	NA	1.22	Allopatry
D. virilis - D. littoralis	0.682291667	0	1.22	Allopatry
D. yakuba - D. erecta	0.994505495	NA	1.4	Sympatry
D. yakuba - D. orena	1	NA	1.12	Sympatry
D. yakuba - D. santomea	0.842032011	0.5	0.3	Sympatry
D. yakuba - D. teissieri	0.988372093	NA	0.39	Sympatry

Supplementary Table 5: Information on reproductive isolation (pre- and postzygotic isolation), Nei's *D* based on allozymes and range overlap classifications for every pair considered in the analysis. These data are taken from Yukilevich (*3*, *4*).

Pair	IM vs SI (Difference in logL -- No Recombination)	IM vs SI (Difference in logL -- With Recombination)
D. affinis - D. athabasca	-0.154304	9.4760575
D. ambigua - D. obscura	-0.63247475	1.8216665
D. ambigua - D. persimilis	-6.669142	0.22948725
D. ambigua - D. pseudoobscura	-7.86996125	0.1155895
D. americana - D. montana	0.1722205	0.9437255
D. americana - D. virilis	-0.00029675	1.1008325
D. americana - D. lacicola	-5.675239	0.293779
D. americana - D. littoralis	0.061838	0.318727
D. arizonae - D. mojavensis	-2.12492575	2.507734
D. auraria- D. rufa	-5.53448175	1.8336325
D. bipectinata - D. malerkotliana	-0.14979975	7.444685
D. bipectinata - D. parabipectinata	0.6888265	2.85252625
D. bipectinata - D. pseudoananassae	0.39919825	10.011253
D. borealis - D. lacicola	-0.582845	3.011703
D. borealis - D. littoralis	-6.40894375	0.89922525
D. borealis - D. montana	-1.304686	1.46773075
D. erecta - D. orena	0.15642725	0.30473525
D. flavomontana- D. montana	-1.47105625	0.28318675
D. insularis - D. willistoni	-6.79094175	1.4804645
D. jambulina - D. punjabiensis	0.18039175	9.0430455
D. kanekoi - D. ezoana	0.491416	4.520206
D. kanekoi - D. littoralis	-6.95620425	1.3461545
D. kanekoi - D. lummei	0.023294	4.623887
D. kanekoi - D. virilis	-0.000216	0.25911775
D. kepulauna - D. albomicans	-2.70380525	1.392413
D. kikkawai- D. leontia	-5.1257895	0.885859
D. kohkoa - D. albomicans	-0.05141025	8.10915425
D. littoralis - D. ezoana	0.4638655	7.7325425
D. littoralis - D. montana	-4.93153525	0.9080145
D. loweii - D. pseudoobscura	0.1259705	1.475313
D. lummei - D. ezoana	0.19643625	5.336632
D. lummei - D. littoralis	0.08678375	8.1485735
D. lummei - D. virilis	-0.0463755	7.52355625
D. malerkotliana - D. parabipectinata	0.13188	10.61952175
D. malerkotliana - D. pseudoananassae	0.29347375	3.239107

D. mauritiana - D. erecta	0.3344945	0.608184
D. mauritiana - D. orena	-0.00181775	0.22230825
D. mauritiana - D. sechellia	-9.2088235	1.4877295
D. mauritiana - D. teissieri	0.0743675	1.60343475
D. mauritiana - D. yakuba	-0.00088875	0.3945455
D. melanogaster - D. erecta	0.04074125	0.0600005
D. melanogaster - D. orena	-0.000964	-0.00286875
D. melanogaster - D. sechellia	-0.0005805	0.4052805
D. melanogaster - D. simulans	0.7517305	0.68122625
D. melanogaster - D. teissieri	0.02389	1.208034
D. melanogaster - D. yakuba	-1.30E-05	0.07559225
D. miranda - D. persimilis	0.4444185	1.53889375
D. miranda - D. pseudoobscura	-0.0001285	1.50660875
D. nasuta - D. albomicans	-1.63932875	1.9127715
D. novamexicana - D. lacicola	-1.455242	4.48309925
D. novamexicana - D. littoralis	-2.22167625	1.75211425
D. novamexicana - D. montana	-5.5826495	0.73650975
D. novamexicana - D. virilis	-4.97667175	0.451082
D. obscura - D. tristis	0.1502035	6.021555
D. parabipectinata - D. pseudoananassae	0.5475705	5.42126225
D. persimilis - D. bifasciata	-5.97366225	3.8885565
D. persimilis - D. pseudoobscura	0.3772995	0.52908975
D. persimilis - D. subobscura	-0.00042525	2.67E-05
D. pseudoobscura - D. bifasciata	-1.89383775	0.03296825
D. pseudoobscura - D. subobscura	0.0205525	0.77406975
D. sechellia - D. erecta	0.106349	0.06987925
D. sechellia - D. orena	-0.00011025	0.229013
D. sechellia - D. teissieri	0.01147	0.075828
D. sechellia - D. yakuba	0.016072	0.1990405
D. simulans - D. erecta	-0.00114175	1.106863
D. simulans - D. orena	-0.00078075	0.80872725
D. simulans - D. teissieri	-0.00128975	1.1766165
D. simulans - D. yakuba	-0.00290275	0.72976775
D. subobscura - D. ambigua	0.29490925	0.79274025
D. subobscura - D. bifasciata	-7.03964625	0.2769065
D. subobscura - D. obscura	0.009463	0.18738925
D. subobscura - D. tristis	-4.9943265	1.55378425
D. sulfurigaster albostrigata - D. kepulauna	-5.10E-05	0.66901075
D. sulfurigaster albostrigata - D. pulaua	-3.273116	1.75750475

D. sulfurigaster albostrigata - D. sulfurigaster bilimbata	-3.31889625	1.5914515
D. sulfurigaster albostrigata - D. sulfurigaster neonasuta	-2.75170775	1.70006225
D. sulfurigaster albostrigata - D. sulfurigaster sulfurigaster	-1.8848695	0.381667
D. sulfurigaster bilimbata - D. pulaua	-1.76603975	1.33646125
D. sulfurigaster bilimbata - D. sulfurigaster neonasuta	-2.7599	2.82219175
D. sulfurigaster bilimbata - D. sulfurigaster sulfurigaster	-0.1085505	3.0562085
D. sulfurigaster neonasuta - D. pulaua	-5.3976675	2.4274
D. sulfurigaster neonasuta - D. sulfurigaster sulfurigaster	-0.73185425	0.7612595
D. sulfurigaster sulfurigaster - D. pulaua	-1.39609025	1.44585225
D. teissieri - D. orena	-0.00012325	0.31169375
D. teissieri- D. erecta	0.0013285	0.34871325
D. triauraria - D. rufa	-1.820373	1.352234
D. virilis - D. ezoana	0.0004845	5.23917
D. virilis - D. lacicola	-2.8273075	0.8447935
D. virilis - D. littoralis	-7.478602	1.587921
D. yakuba - D. erecta	-1.25E-06	0.9683835
D. yakuba - D. orena	-0.00040575	0.36649375
D. yakuba - D. santomea	-0.0001785	0.0026815
D. yakuba - D. teissieri	-6.107981	1.13633825

Supplementary Table 6: Simulation inference. Difference in log likelihood for simulations without and with recombination. For each pair, we simulated a strict isolation with optimized parameters and with the same pair-specific block sizes as the observed dataset. We simulated blocks with and without realistic recombination within blocks and performed model fitting to determine the potential effect of intra-block recombination on our inference. We compare the support for an IM model over the SI model. Values shown are the differences in logL between SI and IM model for each pair in simulations without and with recombination under a strict isolation model.

References and Notes

1. J. A. Coyne, H. A. Orr, Patterns of Speciation in Drosophila. *Evolution*, doi: 10.2307/2409213 (1989).
2. J. A. Coyne, H. A. Orr, " PATTERNS OF SPECIATION IN DROSOPHILA " REVISITED. **51**, 295–303 (1997).
3. R. Yukilevich, Asymmetrical patterns of speciation uniquely support reinforcement in drosophila. *Evolution* **66** (2012).
4. R. Yukilevich, The rate test of speciation: Estimating the likelihood of non-allopatric speciation from reproductive isolation rates in drosophila. *Evolution* **68**, 1150–1162 (2014).
5. P. Nosil, Degree of sympatry affects reinforcement in drosophila. *Evolution* **67** (2013).
6. D. J. Funk, P. Nosil, W. J. Etges, Ecological divergence exhibits consistently positive associations with reproductive isolation across disparate taxa. *Proc. Natl. Acad. Sci. U. S. A.* **103**, 3209–3213 (2006).
7. M. Turelli, J. R. Lipkowitz, Y. Brandvain, On the coyne and orr-igin of species: Effects of intrinsic postzygotic isolation, ecological differentiation, x chromosome size, and sympatry on drosophila speciation. *Evolution* **68** (2014).
8. B. Y. Kim, J. R. Wang, D. E. Miller, O. Barmina, E. Delaney, A. Thompson, A. A. Comeault, D. Peede, E. R. R. D'agostino, J. Pelaez, J. M. Aguilar, D. Haji, T. Matsunaga, E. E. Armstrong, M. Zych, Y. Ogawa, M. Stamenković-Radak, M. Jelić, M. S. Veselinović, M. Tanasković, P. Erić, J. J. Gao, T. K. Katoh, M. J. Toda, H. Watabe, M. Watada, J. S. Davis, L. C. Moyle, G. Manoli, E. Bertolini, V. Košťál, R. S. Hawley, A. Takahashi, C. D. Jones, D. K. Price, N. Whiteman, A. Kopp, D. R. Matute, D. A. Petrov, Highly contiguous assemblies of 101 drosophilid genomes. *eLife* **10** (2021).
9. F. A. Simão, R. M. Waterhouse, P. Ioannidis, E. V. Kriventseva, E. M. Zdobnov, BUSCO: Assessing genome assembly and annotation completeness with single-copy orthologs. *Bioinformatics* **31**, 3210–3212 (2015).
10. T. Brůna, K. J. Hoff, A. Lomsadze, M. Stanke, M. Borodovsky, BRAKER2: Automatic eukaryotic genome annotation with GeneMark-EP+ and AUGUSTUS supported by a protein database. *NAR Genomics Bioinforma.* **3**, 1–11 (2021).
11. S. Neph, M. S. Kuehn, A. P. Reynolds, E. Haugen, R. E. Thurman, A. K. Johnson, E. Rynes, M. T. Maurano, J. Vierstra, S. Thomas, R. Sandstrom, R. Humbert, J. A. Stamatoyannopoulos, BEDOPS: High-performance genomic feature operations. *Bioinformatics* **28** (2012).
12. A. R. Quinlan, I. M. Hall, BEDTools: A flexible suite of utilities for comparing genomic features. *Bioinformatics*, doi: 10.1093/bioinformatics/btq033 (2010).
13. H. Li, Aligning sequence reads, clone sequences and assembly contigs with BWA-MEM. **00**, 1–3 (2013).
14. V. Md, S. Misra, H. Li, S. Aluru, "Efficient architecture-aware acceleration of BWA-MEM for multicore systems" in *Proceedings - 2019 IEEE 33rd International Parallel and Distributed Processing Symposium, IPDPS 2019* (2019).
15. A. Tarasov, A. J. Vilella, E. Cuppen, I. J. Nijman, P. Prins, Sambamba: fast processing of NGS alignment formats. *Bioinformatics* **31**, 2032–2034 (2015).
16. Github, Picard toolkit. *Broad Inst. GitHub Repos.* (2019).
17. J. Köster, S. Rahmann, Snakemake-a scalable bioinformatics workflow engine. *Bioinformatics* **28** (2012).
18. H. Li, A statistical framework for SNP calling, mutation discovery, association mapping and population genetical parameter estimation from sequencing data. *Bioinformatics* **27** (2011).
19. P. Danecek, J. K. Bonfield, J. Liddle, J. Marshall, V. Ohan, M. O. Pollard, A. Whitwham, T. Keane, S. A. McCarthy, R. M. Davies, H. Li, Twelve years of SAMtools and BCFtools. *GigaScience* **10** (2021).
20. D. R. Laetsch, G. Bisschop, S. H. Martin, S. Aeschbacher, D. Setter, K. Lohse, Demographically explicit scans for barriers to gene flow using gIMble. *PLOS Genet.* **19**, e1010999 (2023).
21. P. R. Haddrill, B. Charlesworth, D. L. Halligan, P. Andolfatto, Patterns of intron sequence evolution in Drosophila are dependent upon length and GC content. *Genome Biol.* **6** (2005).
22. P. Andolfatto, Adaptive evolution of non-coding DNA in Drosophila. *Nature* **437** (2005).

23. D. L. Halligan, P. D. Keightley, Ubiquitous selective constraints in the Drosophila genome revealed by a genome-wide interspecies comparison. *Genome Res.* **16**, 875–884 (2006).
24. H. M. Wilkinson-Herbots, The distribution of the coalescence time and the number of pairwise nucleotide differences in the "isolation with migration" model. *Theor. Popul. Biol.*, doi: 10.1016/j.tpb.2007.11.001 (2008).
25. H. M. Wilkinson-Herbots, The distribution of the coalescence time and the number of pairwise nucleotide differences in a model of population divergence or speciation with an initial period of gene flow. *Theor. Popul. Biol.* **82**, 92–108 (2012).
26. Y. Wang, P. McNeil, R. Abdulazeez, M. Pascual, S. E. Johnston, P. D. Keightley, D. J. Obbard, Variation in mutation, recombination, and transposition rates in *Drosophila melanogaster* and *Drosophila simulans*. *Genome Res.* **33**, 587–598 (2023).
27. F. Baumdicker, G. Bisschop, D. Goldstein, G. Gower, A. P. Ragsdale, G. Tsambos, S. Zhu, B. Eldon, E. C. Ellerman, J. G. Galloway, A. L. Gladstein, G. Gorjanc, B. Guo, B. Jeffery, W. W. Kretzschumar, K. Lohse, M. Matschiner, D. Nelson, N. S. Pope, C. D. Quinto-Cortés, M. F. Rodrigues, K. Saunack, T. Sellinger, K. Thornton, H. Van Kemenade, A. W. Wohns, Y. Wong, S. Gravel, A. D. Kern, J. Koskela, P. L. Ralph, J. Kelleher, Efficient ancestry and mutation simulation with msprime 1.0. *Genetics* **220**, iyab229 (2022).
28. J. Kelleher, A. M. Etheridge, G. McVean, "Efficient coalescent simulation and genealogical analysis for large sample sizes" (preprint, Evolutionary Biology, 2015); https://doi.org/10.1101/033118.
29. R. R Core Team, R: A language and environment for statistical computing. (2013).
30. A. Kopp, O. Barmina, Evolutionary history of the *Drosophila bipectinata* species complex. *Genet. Res.* **85**, 23–46 (2005).
31. Kim, B.Y., Gellert, H.R., Church, S.H., Suvorov, A., Anderson, S.S., Barmina, O., Beskid, S.G., Comeault, A.A., Crown, K.N., Diamond, S.E. and Dorus, S., 2024. Single-fly genome assemblies fill major phylogenomic gaps across the Drosophilidae Tree of Life. PLoS Biology, 22(7), p.e3002697.
